# The completed genome sequence of the pathogenic ascomycete fungus *Fusarium graminearum*

**DOI:** 10.1186/s12864-015-1756-1

**Published:** 2015-07-22

**Authors:** Robert King, Martin Urban, Michael C. U. Hammond-Kosack, Keywan Hassani-Pak, Kim E. Hammond-Kosack

**Affiliations:** Department of Computational and Systems Biology, Rothamsted Research, Harpenden, Herts AL5 2JQ UK; Department of Plant Biology and Crop Science, Rothamsted Research, Harpenden, Herts AL5 2JQ UK

**Keywords:** *Gibberella zeae*, Fusarium head blight disease, Fungal centromere sequences, Fungal telomere sequences, Fungal virulence gene hotspots, Completed Sordariomycetes genome

## Abstract

**Background:**

Accurate genome assembly and gene model annotation are critical for comparative species and gene functional analyses. Here we present the completed genome sequence and annotation of the reference strain PH-1 of *Fusarium graminearum*, the causal agent of head scab disease of small grain cereals which threatens global food security. Completion was achieved by combining (a) the BROAD Sanger sequenced draft, with (b) the gene predictions from Munich Information Services for Protein Sequences (MIPS) v3.2, with (c) de novo whole-genome shotgun re-sequencing, (d) re-annotation of the gene models using RNA-seq evidence and Fgenesh, Snap, GeneMark and Augustus prediction algorithms, followed by (e) manual curation.

**Results:**

We have comprehensively completed the genomic 36,563,796 bp sequence by replacing unknown bases, placing supercontigs within their correct loci, correcting assembly errors, and inserting new sequences which include for the first time complete AT rich sequences such as centromere sequences, subtelomeric regions and the telomeres. Each of the four *F. graminearium* chromosomes was found to be submetacentric with respect to centromere positioning. The position of a potential neocentromere was also defined. A preferentially higher frequency of genetic recombination was observed at the end of the longer arm of each chromosome. Within the genome 1529 gene models have been modified and 412 new gene models predicted, with a total gene call of 14,164. The re-annotation impacts upon 69 entries held within the Pathogen-Host Interactions database (PHI-base) which stores information on genes for which mutant phenotypes in pathogen-host interactions have been experimentally tested, of which 59 are putative transcription factors, 8 kinases, 1 ATP citrate lyase (ACL1), and 1 syntaxin-like SNARE gene (GzSYN1). Although the completed *F. graminearum* contains very few transposon sequences, a previously unrecognised and potentially active gypsy-type long-terminal-repeat (LTR) retrotransposon was identified. In addition, each of the sub-telomeres and centromeres contained either a LTR or MarCry-1_FO element. The full content of the proposed ancient chromosome fusion sites has also been revealed and investigated. Regions with high recombination previously noted to be rich in secretome encoding genes were also found to be rich in tRNA sequences. This study has identified 741 *F. graminearum* species specific genes and provides the first complete genome assembly for a Sordariomycetes species.

**Conclusions:**

This fully completed *F. graminearum* PH-1 genome and manually curated annotation, available at Ensembl Fungi, provides the optimum resource to perform interspecies comparative analyses and gene function studies.

**Electronic supplementary material:**

The online version of this article (doi:10.1186/s12864-015-1756-1) contains supplementary material, which is available to authorized users.

## Background

The acquisition of a completed genomic reference sequence has numerous benefits over a near or partial completed sequence. Fully completed genomes have higher impact in terms of the comparative power with other species to evaluate the divergence of pathways and the expansion and contraction of gene families. Completed genomes can also be used to align syntenic regions between related species and thereby improve the accuracy and efficacy of other genome assembly projects [1]. In addition, comparative gene finding tools can be used to annotate other related species [2] and define species specific genes.

The Fusaria are globally important ascomycete fungi of growing agricultural, horticultural, medical and ecological importance. Different species of Fusaria can infect and cause disease on a wide diversity of host species including cereal and non-cereal crops, for example *F. graminearum*, only non-cereal crops (*F. oxysporum* spp and *F. solani*), forestry trees (*F. circinatum*), turf grass (*Microdochium nivale)*, animals, fish, shell fish and humans (*F. solani* species complex). Whereas other Fusarium species are a major source of a high value edible protein consumed by humans (*F. venenatum*) or are used to produce specific biomolecules, for example plant growth promoting gibberellins (*F. fujikuroi*). As a consequence over the past decade, multiple Fusarium sequencing projects have taken place [3, 4] or are currently in progress [5]. Although the haploid Fusarium genomes are small in size, typically 35-50 Mbp, for no Fusarium species is the complete genome sequence available. This is counter-intuitive to providing high quality genomic resources for research however a full spectrum of genomic resources currently exists. This is problematic for the international Fusarium community. The most complete genomic reference sequence of the Fusaria clade is available for *F. graminearum.* The assembled contigs/supercontigs for this species have been assigned to four large chromosomes by using information from a genetic map [4]. But the assembled genome 10 years later still contains > 212,843 unknown bases (N’s), and lacks telomeric and centromeric sequences. Although incomplete this genome has provided the basis to align and assemble genomes of four other plant pathogenic Fusarium species, namely *F. fujikuroi* B14 [6], *F. verticillioides*, *F. oxysporum*, *f. sp lycopersici* [7], *F. pseudograminearum* CS3096, and isolates of *F. pseudograminearum* [8] including the highly virulent *F. pseudograminearum* CS3005 isolate [9]. Unfortunately, each of these other genomes also consists of multiple scaffolds which are heavily interspersed with unknown bases. Consequently their gene annotations may be incomplete. The value of a complete genomic reference sequence is complemented by high-quality annotation of gene and non-coding RNA annotation because all targets of interest can be identified and tested. Gene deletion studies to probe the function of proteins/metabolites potentially linked to virulence or other phenotypic traits of interest [10] are dependent upon the quality of the reference genome and the annotation provided. Therefore an increased confidence in research findings based upon genome and annotation use is observed using a complete reference/annotation versus an incomplete. For example, deletion or silencing of a class of enzymes that are implicated in virulence is not hindered by unknown genes of the same class which maintain the virulence effect.

Some Fusarium species, for example *F. graminearum,* are ideal targets for full genome completion given the predicted low repetitive sequence content and small genome size. Completion of the *F. graminearum* reference genome would be the first completed within the class Sordariomycetes, which contains fungal species with very diverse lifestyles ranging from free living saprophytes, plant invading endophytes, to pathogens of microbes, plants and/or animals. Ongoing comparative analysis between and within Fusarium species is important to food security due to their role in infecting hosts which include; wheat and barley (*F. culmorum* and *F. graminearum*), maize and sorghum (*F. verticillioides),* tomato, tobacco, legumes, cucurbits, sweet potatoes and banana (*F. oxysporum* numerous *formae specialis*), and rice (*F. fujikuroi*).

*Fusarium graminearum* is a fungus of world-wide economic importance because it causes Fusarium head blight (FHB) disease on wheat and barley, also known as head scab disease, and Fusarium stalk and ear rot disease on maize [11]. The infection results in reduced yield affecting grain/kernel mass and quality, and mycotoxin contamination [12, 13], which is a threat to human health [14, 15]. Consequently mycotoxin content in the harvested grain has to be expensively monitored due to maximum tolerated levels of toxins permitted in human foods and animal feeds [16]. Research to reduce FHB disease in cereal species has used the PH-1 strain as a model to identify and understand virulence mechanisms and pathways, with the ultimate aim of developing novel species specific fungicides and/or resistant crop plants [17].

The *F. graminearum* PH-1 strain was originally sequenced using Sanger technology, assembled into 433 contigs and 31 supercontigs, anchored to a genetic map, and annotated by the BROAD institute [4, 18]. Later on the sequence was improved by BROAD, and the gene model annotations were refined by Munich Information Services for Protein Sequences (MIPS) [19, 20], resulting in 13,826 mRNA transcripts in the MIPS annotation (version 3.2). However there remain >397 gaps of unknown sequences within the current assembly represented either by sequential N bases or completely lacking sequence as indicated by the absence of the eight telomeres. In addition, no sequence is available for the four centromeres and 99,079 bp remained unassembled in small contigs. For comparative purposes both the reference and annotation are both referred to in the text as MIPS v3.2.

In this study, to complete the *F. graminearum* genome, whole shotgun re-sequencing of the strain PH-1 to 85-fold coverage was done using short paired end reads followed by *de novo* assembly using various k-mer values. The accompanying annotation was produced by transferring the MIPS v3.2 annotation, then refining the gene model annotation integrating multiple gene prediction algorithms within the Maker2 software [21] as well as by using available RNA-seq from two wild type *F. graminearum* isolates (PH-1 and Gz-3639) to provide additional gene model support. Finally, manual inspection was done on all the gene models. In addition, the PH-1 mitochondrial draft [GenBank:NC_009493] which was 95,676 bp in length was re-evaluated, corrected and reduced to 95,638 bp. To complete this project, the RRes v4.0 genome sequence and the mitochondrial sequence were submitted to European Nuclear Archive (ENA) [EMBL:HG970330, EMBL:HG970331, EMBL:HG970332, EMBL:HG970333, EMBL:HG970334, EMBL:HG970335]. The completed *F. graminearum* genome is available within ENSEMBL fungi [22] and displayed on the plant pathogen specific PhytoPath database [23].

## Results

### Genome closure

#### Assembly

The *de novo* assembly using a sequencing depth of 85x coverage created a *F. graminearum* genome of 36,563,796 bp in length, assembled into four chromosomes and a mitochondrial genome of 95,638 bp in length. There was high accordance between the alignments of the RRes v4.0 genome to the previous MIPS v3.2 reference genome. The alignment of the *de novo* contigs identified a previous mis-assembly at the amino end of chromosome 1 with the 1-306,000 bp sequence observed to be the reverse complement of the true sequence at this position. This was confirmed by the extension at the amino end to a telomere sequence and the manual inspection of mapped reads throughout the genome to detect soft clipping of reads which are indicators of misassemblies. The *de novo* assembly also provided extension of the amino and carboxyl sequence of chromosome 1-4 and identified a common telomere sequence (TAACCC) and reverse complement (GGGTTA) respectively [24] which is found in all vertebrates [25]. This new data completes the four chromosomes at the amino and carboxyl end and confirms the new orientation of the amino terminus of chromosome 1. In addition, a total of 25,707 N bases have been removed to create the new assembly.

**Fig. 1:**
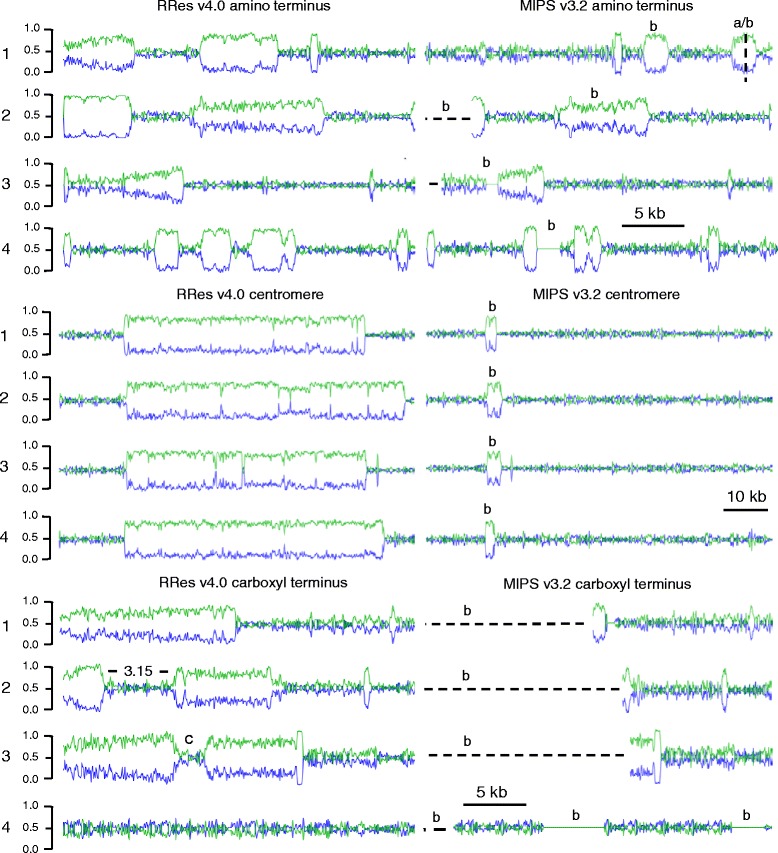
Visual representation of AT rich and repeat rich regions in the RRes and MIPS references. All values are approximations to the nearest 1 kb and images have been scaled to equivalency. The green line represents the AT percentage and the blue the CG percentage to give a combined total of 100 %. The labelling is in the format of a number which represents the chromosome i.e. 1 = chromosome 1, a = the end of a reverse complement section, and b denotes regions in the MIPS version that were extended in the RRes version. The 3.15 label represents where the supercontig 3.15 was placed and “c” represents where FGRRES_20327 is located. The amino terminus set (5’) (upper panel) reveals the AT rich extensions of Chromosomes 2-4 and the sequence that was reverse complemented in Chromosome 1. The centromere set (middle panel) shows chromosome 1 AT rich region extension from 3 kbp to 57 kbp MIPS to RRes respectively within 8.97 Mbp region, Chromosome 2 extension from 3 kbp to 65 kbp MIPS to RRes respectively within 3.27 Mbp region, Chromosome 3 extension from 4 kbp to 56 kbp MIPS to RRes respectively within 5.35 Mbp region, and Chromosome 4 extension from 2 kbp to 61 kbp MIPS to RRes respectively within 5.0 Mbp region. The carboxyl terminus set (lower panel) shows Chromosome 1 extension at the carboxyl end of 14 kbp, Chromosome 2 extension at the carboxyl end of 17 kbp, Chromosome 3 extension at the carboxyl end of 19 kbp, Chromosome 4 extension at the carboxyl end of a repetitive sequence 1.3 Mbp (complete sequence length not shown)

Three previously constructed supercontigs namely 3.26, 3.31, and 3.15 had a similar depth of coverage (~85) to the reads that mapped to the remainder of the genomic reference (Table 1). Therefore each of these sequences became the targets for amalgamation into chromosomes 1 to 4 sequences. After the alignment of contigs from the *de novo* assemblies and replacement of unknown bases these three supercontigs were successfully placed into the genome. Supercontig 3.15 was placed at the carboxyl end of chromosome 2 (Fig. 1) to provide the subtelomeric AT-rich sequence and the missing telomere sequence. Supercontigs 3.26 and 3.31 were placed respectively in chromosomes 3 at position 2,466,775-2,469,779 in which a new gene prediction is found (FGRRES_20327), and chromosome 1 at position 4,541,855-4,543,915.

**Table 1:** Chromosome and Supercontig sequence coverage of the *F. graminearum* PH-1 isolate and MIPS reference. Identification of underrepresented repeating sequences represented in the MIPS reference sequence could be calculated by dividing the total corrected average observed coverage by the original sequencing depth of 85.

MIPS Chromosome/Supercontig number	Length (bp)	Raw average coverage^b^	Bases with coverage (%)^c^	Corrected average coverage^d^	Calculated multiples of supercontig sequences in genome^e^
1	11,694,295	79.5	100.0	79.5	1
2	8,911,601	90.7	99.6	90.3	1
3	7,711,129	90.4	95.5	86.3	1
4	8,029,942	70.6	91.8	64.8	1
4: 7,953,943+ bp^a^	75,973	1,000.2	77.8	778.16	9
3.13	12,585	1,083	96	1,039.7	12
3.18	8,774	930.3	99	921	11
3.2	7,037	1,000.6	91.3	913.5	11
3.29	2,326	731.9	95	695.3	8
3.28	2,628	687.8	98.1	674.7	8
3.3	2,172	573.5	99.2	568.9	7
3.22	6,062	573.5	75.5	433	5
3.24	6,000	557.4	73.5	409.7	5
3.23	6,331	541.4	74.2	401.7	5
3.21	7,489	551.3	78.2	431.1	5
3.25	6,524	368.1	63	231.9	3
3.15	10,471	96.7	100	96.7	1
3.31	2,080	85.8	100	85.8	1
3.26	2,996	95.7	100	95.7	1
3.12	15,604	438.9	99.4	436.3	5

^a^Chromosome 4 from 7,953,943 bp onwards, this is the start of the repeating RNA annotated sequence

^b^The read depth observed across the length of the sequence

^c^Percentage of bases that are A,C,T,G but not N

^d^The calculated N bp corrected coverage

^e^The number of copies of the sequence that should be represented in the reference sequence

Chromosome 4 was found to lack a telomere sequence at the carboxyl terminus but was found instead to possess a region of highly repetitive sequences solely annotated as rRNA coding. This large feature of the genome was found to extend for at least 1.38 Mbp and was identified using the following manual curation procedure. Mapping the reads of the re-sequenced PH-1 isolate to the MIPS reference sequence revealed a disproportionately higher read coverage for 12 of the 15 supercontigs not yet placed within the genome compared to the rest of the *de novo* assembled genome (Table 1). This suggested that some or all of these supercontig sequences were represented in multiple copies. The high copy number of each supercontig may have contributed to the difficulty experienced in prior efforts to place these sequences within chromosomes 1-4 [4]. Following manual inspection, ten of these repetitive sequences containing supercontigs could be aligned to the carboxyl end of chromosome 4. Each of these supercontigs contained rRNA encoding repetitive sequence. This manual procedure permitted the chromosome 4 to be extended by 1.38 Mbp at the carboxyl terminus by further extrapolation of rRNA sequence by division of the total mapped read coverage of this region by the expected 85 x coverage. Supercontig 3.13 was identified as mitochondrial DNA and was transferred into the mitochondrial assembly pipeline. The unknown sequence within supercontig 3.12 (MIPS length 15,604 bp) was corrected and the only supercontig remaining which had multiple copy coverage (>85) but did not align partially or completely to chromosome 1-4 sequences or the mitochondria. Manual curation of the mapped reads suggests this supercontig to be either a repeating unit or a circular sequence of 5846 bp with no known genomic loci. Consequently this supercontig was reduced to one single repeating unit. The identification of genomic loci of the remaining supercontigs from the original Sanger sequencing and unknown sequence replacement completes chromosomes 1-4 sequences.

The remaining 1.28 million re-sequenced un-mapped reads underwent *de novo* assembly of which the resulting 777,064 remaining reads and 79,424 contigs were analysed using BLAST with a 0.01 significance value followed by MEGAN [26]. These reads and contigs were shown to represent contamination (99.1 % data not shown) with 74 sequences that had fungal hits. The length of these 74 individual contigs did not exceed 400 bp and the concatenated length was 13,850 bp. The 55,093 unplaced contigs from the original Sanger sequencing used in the MIPS v3.2 genome were mapped to the new reference and mitochondrial sequence, RRes v4.0. The majority mapped to these sequences using BWA MEM with only 3131 sequences remaining to be placed of a combined length of 460,949 bp. These remaining sequences were assigned using BLAST with a 0.01 significance value and MEGAN to Mouse 55,653 bp (90 sequences), low complexity 186,753 bp (404 sequences), no hits 193,456 bp (2375 sequences), and not assigned 25,087 bp (262 sequences). The BLAST and MEGAN analysis of the remaining sequencing data from both sequencing platforms suggests that there are no additional chromosomes to the four represented in RRes v4.0.

### The completed *F. graminearum* genome

The revised RRes v4.0 of the *F. graminearum* PH-1 strain genomic reference, deposited to ENA, contains chromosomes 1 through 4 and comprises 36,563,796 bp with 12 ‘N’ bases. This number excludes the significantly extended repetitive rRNA encoding region at the carboxyl end of chromosome-4 (Table 2). In contrast in the MIPS v3.2 genome, chromosomes 1-4, comprises 36,261,856 bp with 25,707 ‘N’ bases whilst the BROAD FG3.0 version is in the form of multiple large supercontigs 3.1-3.10 and comprises 36,073,610 bp (supercontigs above 25 kb) with 210,520 ‘N’ bases [5]. The genomic length of RRes v4.0 chromosomes 1-4 is 354,665 bp longer than that of MIPS v3.2. In order that this figure was not distorted, the calculations excluded the repetitive rRNA annotated supercontigs within MIPS and complementary sequence at the carboxyl end of chromosome 4 in both the MIPS and RRes v4.0 reference because of significant extension in RRes v4.0. The 12 unknown bases in the RRes v4.0 are found at a single poly-A locus within the centromere of chromosome 1. There were 2,433 homozygous mutations observed when mapping the re-sequenced PH-1 to the MIPS v3.2 genomic reference consisting of: 2,025 snps with a transition/transversion ratio of 0.5555, 226 insertions, and 182 deletions. These mutations may have arisen due to strain passaging or a consequence of sequencing errors from the original sequencing but the observation of these mutations clustering at loci suggests due to passaging. Readers are reminded that all genomic reference sequences are snapshots of a individual or a subset of individuals of a population. The size of the four chromosomes was found to be; Chromosome 1: 11,760,938 bp (excluding 12 ‘N’ bases), Chromosome 2: 8,997,558 bp, Chromosome 3: 7,792,947 bp, and Chromosome 4: 9,407,501 bp.

**Table 2:** Comparative statistics on the length and nucleotide composition of the four *F. graminearum* chromosomes. Comparison of the MIPS (old) and RRes (new) versions of the reference genome

Reference	MIPS			RRes			
Chromosome	Number of total bases (bp)	Number of ‘N’ bases (bp)	Total number of bases minus ‘N’ bases (bp)	Number of total bases (bp)	Number of ‘N’ bases (bp)	Total number of bases minus ‘N’ bases (bp)	Length difference RR vs. MIPS (bp)^a^
1	11,694,295	65,691	11,628,604	11,760,950	12	11,760,938	+132,334
2	8,911,601	49,430	8,862,171	8,997,558	0	8,997,558	+135,387
3	7,711,129	32,005	7,679,124	7,792,947	0	7,792,947	+113,823
4	8,029,942 (85,155^b^)	65,717	7,964,225	9,397,177 (1,384,836^b^)	0	9,407,501	+1,432,952
Totals	36,346,967	212,843	36,134,124	37,948,632	12	37,958,944	+1,824,820

^a^Number of total bases minus ‘N’ bases (bp) used in difference comparison

^b^Approximate total length of repeating sequence at the carboxyl end of chromosome 4

To complete the *F. graminearum* mitochondrial genome, 15 SNPs were corrected, 28 indels deleted and 2 indels were inserted. As a result, the new mitochondrial reference genome deposited at ENA has a length that is slightly lesser in size at 95,638 bp, compared to the original size of 95,676 bp. Both the original Sanger sequence reads and the new mitochondrial genome obtained by paired end reads exhibited no polymorphisms. This result not only confirms the reliability of the new version of the genome, but clearly demonstrates that there has been no change in the mitochondrial sequence of strain PH-1 in over a decade of repeated fungal growth *in vitro*, *in planta* and long term storage at -80C.

### Resolving two specific genome features: telomeres and centromeres

The placement and extension of AT rich sequence is normally difficult to resolve during genome assembly due to repetitive DNA content. These sequences predominate at the amino and carboxyl ends of each chromosome and represent the subtelomeric regions. In most eukaryotic species there are multiple such regions within the relative vicinity of the repeating telomere sequence (TAACCC). This was found to be the case in the seven assembled subtelomeric regions. In Fig. 1, the original and de novo extended AT rich sequences at the end of each chromosome are depicted. To achieve the subtelomeric assembly, a multi k-mer approach was used in which a range of values from 61 to 85 was sequentially aligned to extend the sequence. However, this difficult part of the assembly was most likely achieved because of the previously noted intrinsically low abundance of repeat sequences in *F. graminearum* PH-1 [4].

The extension of an AT rich region within each chromosome permitted the four centromere regions to be defined and accurately positioned. In chromosome 1, an AT region starting at 8.97 Mbp was extended from 3 kbp to 57 kbp, in chromosome 2 at 3.27 Mbp a 3 kbp region was extended to 65 kbp, in chromosome 3 at 5.35 Mbp a 4 kbp region was extended to 56 kbp, and in chromosome 4 at 5.0 Mbp a 2 kbp region was to 61 kbp. The centromere position quoted is directly related to the AT rich positions originally noted in the MIPS version, see Fig. 1. These results indicate that each centromere has an asymmetric position in each chromosome. Therefore each of the four *F. graminearum* chromosomes is submetacentric with respect to centromere positioning. The evidence supporting these regions are centromere sequences was tested by the Connolly et al. study [27]. Where there was centromeric sequence present, which was only in chromosome 3 in the MIPS version of the reference, then H3K9me3 binding was observed but no H3K27me3, see Additional file 1.

### Annotation of the completed *Fusarium graminearum*

#### The revised gene call

From a total of 13,826 mRNA transcripts presented in the MIPS v3.2 genome most were transferred to the RRes v4.0 genome. However, sixteen were deleted due to sequence changes, two were redundant isoforms and removed (see Additional file 2), 63 gene models were removed due to merging and five gene models were split. In addition, gene models that had alternative model predictions which were longer were explored using InterProscan5 and manually corrected where applicable. The gene models that either did not 100 % match after transfer or had been revised by extension, splitting or merging have been given the ID extension “_M”, representing modification. Some of the most common types of changes made are described in the following three generic examples: (1) An extended or reduced gene, for example, FGSG_ 17172 (hereafter referred to only as 17172 would become 17172_M, (2) splitting of a gene into two ORFs results in the new gene designations 17172_A_M and 17172_B_M, and (3) joining of a gene with other gene models i.e. 17172 and 17173, was designated as 17172_3_M keeping only the unique digit for the second gene if sequential ID’s or if not sequential then the full gene ID for each was used i.e. 17172 and 16222 becomes 17172_16222_M. These changes to the gene names were done to signify that these gene models had been changed from the previous MIPS v3.2 annotation but this approach also ensured the retention of the original gene ID. In total, 1529 modified gene models are presented in the RRes v4.0 genome annotation. Also, 412 new gene models have been predicted of which 152 have supporting RNA-seq evidence from cultured mycelium or spore transcriptome assemblies. These have arisen because of the replacement of the ‘N’ sequences or alternative gene prediction software. The new gene models have been given sequential numbers from 20,000, beginning at the amino end of chromosome-1 and ending at the carboxyl end of chromosome-4. The Maker annotation software was used to transfer gene annotation ID’s, predict gene models to correct MIPS gene models v3.2 and add additional new genes using as evidence, RNA-seq for the mycelium and spores of PH-1 [EMBL:SRP039087], Z-3639 mycelia harvested 24 h after inoculation of wild-type conidia in complete medium RNA-seq [EMBL:SAMN02400310], proteins from Uniprot from Fusarium with evidence, and Cogeme EST transcripts [28]. All evidence and final gene model predictions are available through the Ensembl fungi tracks as “Fusarium graminearum”. The total number of gene models identified in the completed *F. graminearum* genome is 14,164 which is in contrast to 13,322 and 13,826 in the BROAD and MIPS v3.2 annotations respectively (see Table 3). A comparison of the length of proteins between the RRes and MIPS v3.2 annotations shows proportionally the total percentage of proteins with a length below 149 aa, and between 150 and 999 aa to increase and decrease respectively for the RRes set (see Additional file 3). This figure is a reflection of the greater proportion of genes that are predicted as a single exon in the RRes v4.0 vs the MIPS v3.2 set (see Additional file 4).

**Table 3:** Basic statistics of the different reference and annotation versions of *F. graminearum.* A comparison of *F. graminearum* genome version statistics between BROAD, MIPS, and RRes

	BROAD	MIPS	RRes
Genome size (bp)^a^	36,565,771	36,553,761	38,060,440
Scaffolds^b^	31	19	5
GC (%) content^c^	48.3	48.3	48.2 (48.0^d^)
Spanned gaps	402	402	1
Predicted Genes	13,322	13,826	14,164
Repetitive (%)	0.24	0.24	0.24
Transposable elements (%)	0.029	0.029	0.060
ENA project accession	PRJNA13839	N/A	PRJEB5475

^a^including all scaffolds, N bases, and the mitochondria

^b^including all scaffolds, N bases excluding the mitochondria

^c^Excluding N’s and mitochondria

^d^Excluding N’s, mitochondria and large repetitive sequence at the carboxyl end of chromosome

### The gene annotation

An analysis of the RRes v4.0 annotation using Blast2GO with the NCBI nr database and InterProscan5 revealed a total of 12,691 genes that were Blast2GO annotated or had blast hits, whilst 5,442 genes have no GO annotation (38.4 %). There are an additional 386 level-2 GO biological process GO annotations and 159 molecular function GO annotations in the RRes v4.0 annotation compared to the MIPS v3.2 (Table 4 and Table 5). The number of proteins with alpha/beta hydrolase fold-1 or Zn(2)-C6 fungal-type DNA-binding domain have increased from 21 to 81 and 316 to 358, respectively (Table 6). The greatest increases for biological processes were in the categories; biological regulation, single-organism process, cellular process, metabolic process, and for molecular functions; catalytic activity, binding, and nucleic acid binding transcription factor activity.

**Table 4:** GO term (level 2) summary of the 412 new gene annotations. GO terms summaries for biological process and molecular function of the 412 new genes annotated in the RRes V4.0 gene set

Biological process		
GO-id	GO-term	No.
GO:0044699	single-organism process	18
GO:0050896	response to stimulus	2
GO:0008152	metabolic process	32
GO:0009987	cellular process	22
GO:0032502	developmental process	2
GO:0071840	cellular component organization or biogenesis	2
GO:0065007	biological regulation	10
GO:0051179	localization	4
GO:0032501	multicellular organismal process	2
GO:0023052	signalling	1
Total		99
Molecular function		
GO-id	GO-term	No.
GO:0005215	transporter activity	3
GO:0001071	nucleic acid binding transcription factor activity	8
GO:0004872	receptor activity	1
GO:0003824	catalytic activity	25
GO:0005488	binding	24
GO:0030234	enzyme regulator activity	1
Total		64

**Table 5:** Level 2 GO terms of the modified gene annotations (_M) between RRes and the MIPS counterparts. GO summaries were extracted using Blast2GO

Biological process				
GO-id	GO-term	RRes No.	MIPS No.	Diff No.
GO:0032501	multicellular organismal process	8	3	5
GO:0048511	rhythmic process	2	1	1
GO:0022610	biological adhesion	7	7	0
GO:0051704	multi-organism process	6	3	3
GO:0051179	localization	165	150	15
GO:0008152	metabolic process	617	539	78
GO:0023052	signalling	30	26	4
GO:0000003	reproduction	4	4	0
GO:0009987	cellular process	482	428	54
GO:0050896	response to stimulus	72	56	16
GO:0065007	biological regulation	152	112	40
GO:0002376	immune system process	1	0	1
GO:0040007	growth	1	1	0
GO:0044699	single-organism process	408	357	51
GO:0032502	developmental process	10	5	5
GO:0022414	reproductive process	3	1	2
GO:0040011	locomotion	1	0	1
GO:0071840	cellular component organization or biogenesis	52	41	11
Total		2021	1734	287
Molecular function				
GO-id	GO-term	RRes	MIPS	Diff
GO:0004872	receptor activity	6	3	3
GO:0060089	molecular transducer activity	7	5	2
GO:0001071	nucleic acid binding transcription factor activity	79	55	24
GO:0005198	structural molecule activity	21	20	1
GO:0005085	guanyl-nucleotide exchange factor activity	2	2	0
GO:0009055	electron carrier activity	5	5	0
GO:0016209	antioxidant activity	5	5	0
GO:0030234	enzyme regulator activity	4	4	0
GO:0005488	binding	514	491	23
GO:0003824	catalytic activity	488	449	39
GO:0005215	transporter activity	75	72	3
Total		1206	1111	95

**Table 6:** InterProscan5 domain annotation comparison between RRes v4.0 versus MIPS v3.2. The number of proteins identified with protein domains associated with InterProscan ID’s that are linked to virulence genes. Those in bold are increased in the RRes v4.0 set

			RRes	MIPS
Parent Inter ID	Child Interpro ID		no. protein	no. protein
IPR017853	Glycoside hydrolase superfamily		122	122
IPR029058	Alpha/Beta hydrolase fold		286	N/A
	IPR000675	cutinase	**13**	12
	IPR001031	thioesterase	5	5
	IPR000383	Xaa-Pro dipeptidyl-peptidase-like domain	4	4
	IPR000073	Alpha/beta hydrolase fold-1	**81**	21
	IPR001375	Peptidase S9, prolyl oligopeptidase, catalytic domain	9	9
	IPR002018	Carboxylesterase, type B	26	26
	IPR002921	Fungal lipase-like domain	8	8
	IPR002925	Dienelactone hydrolase	8	8
	IPR003140	Phospholipase/carboxylesterase/thioesterase	5	5
	IPR013094	Alpha/beta hydrolase fold-3	30	31
	IPR029059	Alpha/beta hydrolase fold-5	12	N/A
IPR003439	ABC transporter-like		62	62
IPR011050	Pectin lyase fold/virulence factor		33	33
	IPR000070	Pectinesterase, catalytic	3	3
IPR004835	Chitin synthase		**12**	9
IPR001138	Zn(2)-C6 fungal-type DNA-binding domain		**358**	316
IPR001128	Cytochrome P450		114	114
IPR011701	Major facilitator superfamily		**250**	248
IPR015433	Phosphatidylinositol kinase		**3**	2
IPR015500	Peptidase S8, subtilisin-related		28	29
IPR001283/IPR014044	Cysteine-rich secretory protein, allergen V5/Tpx-1-related/CAP domain		5	5^a^
IPR011329	Killer toxin		4	4

^a^One of these proteins was only partial

The GO annotation assigned to the new genes are detailed in Table 4 (for full BLAST and GO annotations see Additional file 5). The new genes given enzyme designation codes are listed in Table 7. Some of the new genes potentially have roles in primary metabolism, secondary metabolism or cell signalling. Some newly predicted genes of particular interest due to coding for metabolic pathway enzymes or orthologous in virulence mechanisms are as follows: FGRRES_20327 is predicted to have alpha-N-arabinofuranosidase activity (enzyme code: 3.2.1.55), is located in an average % GC region of approximately 2 kb in length neighboured by two AT rich regions of approximately 9 kb length at the carboxyl end of chromosome 3. FGRRES_20406 and FGRRES_20294 are both serine threonine protein kinases (enzyme code: 2.7.11). FGRRES_20377 is predicted to be a cutinase (enzyme code: 3.1.74). FGRRES_20330 is predicted to be related to the enzyme galactoside o-acetyltransferase (enzyme code: 2.3.1). FGRRES_20264 is predicted to be involved in the inositol phosphate metabolism pathway, (enzyme code: 2.7.1.67). FGRRES_20189 is predicted to be involved in pyruvate metabolism Acetyl-CoA to homocitrate and lysine biosynthesis of 2-oxoglutarate to homocitrate (enzyme code: 2.3.3.14). Finally, FGRRES_20176 has a pathogenicity protein description and GO transporter activity.

**Table 7:** New enzyme’s from the new RRes gene set. New enzyme codes designated to the 412 new RRes V4.0 gene set

Gene ID	Enzyme code	Enzyme Name or description
20327	EC:3.2.1.55	Non-reducing end alpha-L-arabinofuranosidase
20406	EC:2.7.11	Transferring phosphorous-containing groups
20377	EC:3.1.74	Cutinase
20330	EC:2.3.1	Acyltransferases
20264	EC:2.7.1.67	1-phosphatidylinositol 4-kinase
20189	EC:2.3.3.14	Homocitrate synthase
20172	EC:1.14.12	Oxidoreductases
20056	EC:1.14.11	Oxidoreductases
20292	EC:3.4.23	Aspartic endopeptidases
20373	EC:1.14.11	Oxidoreductases
20101	EC:1.1.1.158	UDP-N-acetylmuramate dehydrogenase

An additional 19 new enzymes have been identified from the modified RRes gene model set, see Table 8. In total, 287 new biological process level GO terms have been identified and 95 new molecular function level 2 GO terms were found (Table 5). Of the modified genes, two genes were of particular interest in terms of virulence, FGRRES_17235_M, because this gene originally had no functional annotation but was extended to be identified as coding for a cysteine rich secretory protein, allergen V5/Tpx-1-related with CAP and signal peptide domains, which were previously linked with plant pathogenesis proteins of the PR-1 family [29] and identified in the highly virulent *F. graminearum* strain CS3005 (gene ID: FG05_09548). The second gene 15917_M was identified as a endo-1,4-beta-xylanase enzyme acting on plant cell walls by endohydrolysis of (1- > 4)-beta-D-xylosidic linkages in xylans, also identified as part of the secretome.

**Table 8:** New enzyme codes from the modified RRes gene set. Enzymes codes identified in the modified RRes gene subset (_M) not found in the MIPS annotation

Gene ID	Enzyme code	Enzyme Name
06098_M	EC:6.1.1.20	Phenylalanine--tRNA ligase
08061_M	EC:4.1.1.49	Phosphoenolpyruvate carboxykinase (ATP)
01195_M	EC:3.6.3.8	Calcium-transporting ATPase
07202_M, 11727_M	EC:3.6.1.15	Nucleoside-triphosphate phosphatase
15917_M	EC:3.2.1.8	Endo-1,4-beta-xylanase
16359_B_M	EC:3.1.4.11	Phosphoinositide phospholipase C
10980_M	EC:2.7.7.49	RNA-directed DNA polymerase
13343_M	EC:2.7.1.30	Glycerol kinase
09688_M	EC:2.5.1.18	Glutathione transferase
16945_M	EC:2.3.1.225	Protein S-acyltransferase
13161_M	EC:2.2.1.2	Transaldolase
08613_M	EC:2.1.1.71	Phosphatidyl-N-methylethanolamine N-methyltransferase
15680_M	EC:1.3.1.9	Enoyl-[acyl-carrier-protein] reductase (NADH)
06127_M	EC:1.2.1.2	Formate dehydrogenase
15680_M	EC:1.13.12.16	Nitronate monooxygenase
04922_M	EC:1.1.1.9	D-xylulose reductase
03873_M	EC:1.1.1.158	UDP-N-acetylmuramate dehydrogenase
03648_M	EC:1.1.1.184	Carbonyl reductase (NADPH)

For nine genes from the MIPS v3.2 annotation, which were in regions of unknown sequence an improved level of annotation was possible for one gene. FGRRES_16611 previously classified as a hypothetical protein of 329 amino acid (aa) length but was extended to 437 aa and identified as an UAA transporter. The remaining eight corrected gene models could not be assigned additional functionality.

A total of 504 RNA annotations were found across the four fully assembled chromosomes including 319 tRNA, 62 rRNA, 121 ncRNA, and 2 tmRNA see Table 9. In addition a further 538 rRNA were identified in the extensive repetitive region present at the carboxyl of chromosome 4. A non-coding RNA 4.5S RNA that has sequence similarity to a Plant_SRP (RFAM: RF01855) and Fungi_SRP (RFAM:RF01502) [30] of 1.00 E-4 and 2.00 E-31, which is the only one identified in the genome at position 5,836,584 bp on chromosome 3, may be important to the effectiveness of the secretome and pathogenicity. Signal recognition particles (SRP) are universally conserved ribonucleoproteins that direct the traffic of proteins within the cell allowing them to be secreted [31] and therefore are a potentially important mechanism for virulence.

**Table 9:** The number of coding and non-coding gene models predicted in the RRes and MIPS versions. Summary of coding and non-coding annotations of both the RRes and MIPS annotations across chromosomes 1-4 and supercontigs

Chromosome	RRes gene no.	MIPS gene no.	RNA RRes (total)	tRNA RRes	rRNA RRes	ncRNA RRes	tmRNA RRes
1	4393	4303	118	74	9	34	1
2	3645	3549	156	97	32	27	0
3	3090	2999	121	87	8	25	1
4^a^	3032	2969	99	61	13	25	0
4^b^ (1,384,836 bp)	0	0	538	0	538	0	0
Supercontig_3.12	4	3	10	0	0	10	0
Supercontig_3.15	n/a	3	n/a	0	0	0	0
Total	14,164	13,826	504 (538^a^)	319	62 (538)	121	2

^a^Without carboxyl end repeating units with RNA annotation

^b^carboxyl end repeating units with RNA annotation

To complete the genome annotation, the remaining RRes v4.0 supercontig 3.12 was found to contain four genes including an additional new gene of 34 aa length (FGRRES_ 20410). The only gene to have InterProscan or BLASTP (E < 0.01) annotation was FGRRES_14025 which contains an intron endonuclease, group 1 domain and is a member of the GIY-YIG nuclease superfamily [32], typically found predominantly in phage and mitochondria. This supercontig may represent phage DNA rather than mitochondrial due to the lack of alignment with the mitochondrial DNA. In addition to annotation using four gene predictors, open reading frame annotation of this contig showed no additional proteins that matched within the NCBI database therefore there are unlikely to be any small genes in addition to these four predicted genes.

Deoxynivalenol (DON) produced by *Fusarium graminearum* is a potent B-type trichothecene mycotoxin [33] and virulence factor to disease formation in wheat floral tissue [34]. Commonly found in harvested cereal grains, DON lowers grain quality and is a serious health concern to humans and animals [35]. DON synthesis has been assigned to 11 genes (Tri1FGRRES_00071, Tri3 FGRRES_03534, Tri4 FGRRES_03535, Tri5 FGRRES_03537, Tri7A FGRRES_02115, Tri7B FGRRES_03533, Tri8 FGRRES_03532, Tri101 FGRRES_07896, Tri11 FGRRES_03540, Tri13A FGRRES_03860, Tri13B FGRRES_10629 and four regulators Tri6 FGRRES_16251, TRI10 FGRRES_03538, Tri12 FGRRES_15001, and Tri12 FGRRES_11025 [36]. Changes to gene structures of these and other mycotoxin associated sequences may affect previous or future studies involving gene deletion/gene silencing studies. Within the RRes v4.0 gene annotations the Tri-gene cluster situated on chromosome 2, Tri3 FGRRES_03534_M 15-O-acetyltransferase protein domain has increased from 391 aa to 413 aa.

Other less prevalent mycotoxins produced by *F. graminearum* include the estrogenic polyketide zearalenone and nivalenol (NIV), which is structurally related to DON. The *F. graminearum* PH-1 strain lacks functional copies of the Tri13 and Tri7 genes resulting in the inability to produce NIV. Zearalenone induces hyperoestrogenic responses in mammals and can result in reproductive disorders in farm animals, therefore it is of agricultural importance to limit levels in the harvested grain. Zearalenone synthesis requires four genes in a cluster on chromosome 1, namely FGRRES_15980, FGRRES_17745, FGRRES_15982, and FGRRES_02398 [37]. In the Zearalenone gene cluster, the PS-DH domain of the protein 15980_M has increased from 145 aa to 299 aa and the ketoacy-synt-c domain has increase from 115 aa to 117 aa.

Although the DON production pathway is well studied, the regulatory mechanisms are less well characterised [38] and unknowns such as the oxygenation step involving converting the C8 hydroxyl to a carbonyl group and the deacetylation of C15 have not been assigned an enzyme [39]. A greater understanding of genetic factors regulating toxin biosynthesis could lead to the development of new strategies aimed at reducing toxin accumulation in the host tissue. In the MIPS annotation there are eight gene predictions with aflatoxin biosynthesis regulatory protein annotations with two that have transcription factor domains. Although aflatoxin is not known to be produced by *F. graminearum* there role(s) may be associated with another mycotoxin pathway such as DON. Two modified gene predictions from the RRes v4.0 set that are annotated as aflatoxin biosynthesis regulatory proteins and one that also has a transcription factor domain (FGRRES_12018_M and FGRRES_03294_M respectively) are additions to this small clade of functionally unknown regulatory genes.

### Analysis of the predicted secretome

The secretome of a fungus is the set of secreted proteins that frequently defines and facilitates the host infection process [40]. During a fungal pathogens interaction with a host, the invading fungal cells secrete various proteins that can block host responses, i.e. the fungus *Ustilagomaydis* secretes Cmu1 which can move through plant cells and redirect plant metabolic pathways to favour fungal infection [41], or kill host cells i.e. for *F. graminearum*, 109 genes coding for secreted cell wall degrading enzymes have previously been identified [42]. Characterisation of the secretome is fundamental to the understanding of the mechanisms of host infection and pathogen virulence.

**Fig. 2:**
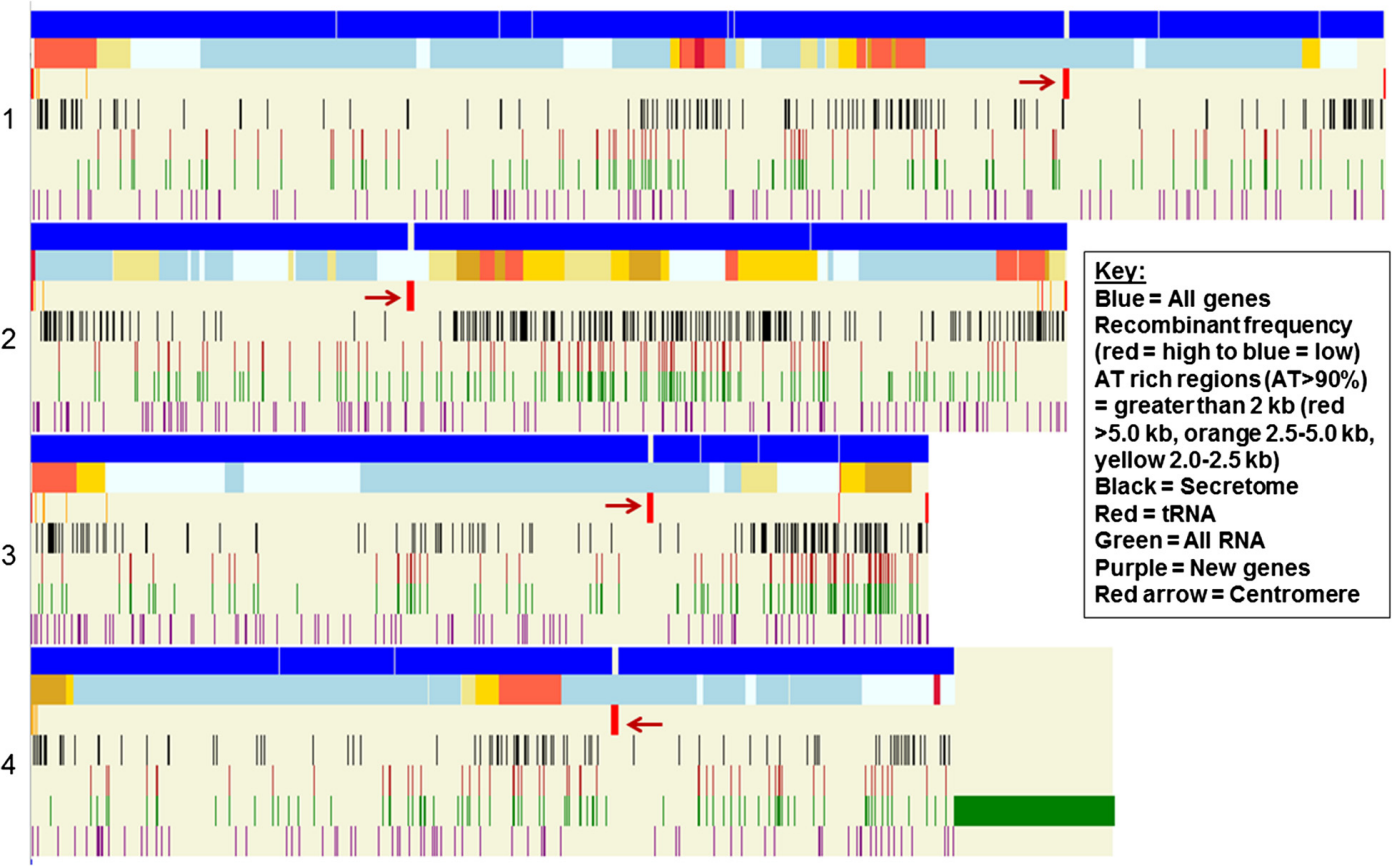
Gene and RNA distribution across the four chromosome (Chr 1–4). Each vertical bar represents a single gene or RNA annotation, aligned next to a heat map for genetic recombination (red = high to blue = low, recombination frequency - second row down of each chromosome). Rows 3 through to rows 7 denote the following: All gene annotations, recombinant frequency, AT rich regions (AT > 90 %) greater than 2 kb (red = >5.0 kb, orange = 2.5-5.0 kb, yellow = 2.0-2.5 kb), predicted secretome (n = 615), tRNA annotations, all RNA annotations, and new gene annotations only. The location of each centromere is highlighted in row 3 with a red arrow

Using the MIPS v3.2 assembly and annotation in 2012, Brown et al. [42] predicted the secretome to contain 574 proteins (575 including a protein without an ‘M’ start codon). Using the new RRes v4.0 predicted protein set and updated versions of the software we obtain 616 proteins using the Brown et al. pipeline to represent the refined secretome (see Additional file 6 for a complete gene ID list and detailed methodology). 144 of the RRes predictions were confirmed using proteomics from the Yang et al. [43] and Paper et al. [44] studies. 30 of the 70 proteins identified in the Yang et al. study from proteins identified in *F. graminearum* culture supernatants after growth in wheat or barley flour-containing medium, and 137 of the 286 from the extracellular proteins *in vitro* and *in planta* from the pathogenic fungus *F. graminearum* identified in the Paper et al. study were confirmed in the in silico predictions. Of the 616 proteins predicted, 55 are from modified genes and 12 are from newly predicted genes. In total, 515 protein ID’s of the newly refined secretome set match the original predictions. Whereas 59 secreted proteins are unique to Brown et al. and 101 unique to RRes v4.0 (see Additional file 7). Of the complete predicted secretome, 119 proteins have some level of annotation whilst 497 proteins lack annotation and 73 proteins (11.85 %) of the newly refined secretome have a cysteine content of > 5 %. Although genomic distribution of the secretome is not altered, an overview diagram of the secretome genes and tRNA annotated genes shows an overlap of clustering, in particular on chromosome 2 and 3, See Fig. 2.

### Analysis of genes predicted to possess nucleic acid binding transcription factor activity

Proteins that bind to specific DNA sequences and regulate transcription of genes are termed transcription factors. They represent an important family of proteins controlling activities of the organism that regulate traits important for growth, development, stress response, pathogenesis, and toxin production, therefore are of importance to the development of fungicides for intervention. Within the RRes v4.0 gene set we have modified 25 gene annotations of the MIPS set and predicted 7 new genes (See Additional file 8). The modified set of gene annotations shows 18 gene models that lacked a Zn(2)-C6 fungal-type DNA-binding domain in the MIPS annotation to have been extended to include it in the RRES v4.0 set, and 15 of these have been identified as having Gal4 protein domain and therefore are potential positive regulators for the gene expression of galactose-induced genes. Of the new set of gene annotations predicted with transcription factor activity: FGRRES_20168 is annotated as having oxidoreductase activity, FGRRES_20126 is annotated as a component of a protein kinase CK2 complex, and FGRRES_20239 is annotated as having lipid glycosylation activity which is part of a family of transferases involved in the final stages of the biosynthesis of antibiotics vancomycin and related chloroeremomycin.

### Genes located in close proximity to the telomere and centromere sequences

The centromeres are the region of the chromosomes that determine kinetochore formations. Each centromere enables pairing of the sister chromatids and interacts with spindle microtubules during mitotic chromatid segregation and homologous chromosome segregation during meiosis. Centromere structure in *F. graminearum* and more generally in eukaryotes is poorly understood [27]. For eukaryotes it was suggested that centromeres may be derived from subtelomeric regions [45]. The four centromeres identified in *F. graminearum* chromosome 1-4 respectively are 56,581, 65,181, 56,387, 60,933 bp long and are marked by their similar high AT content, 87.2, 86.4, 87.2, and 87.9 % respectively. Chromosome 3 is the only chromosome to have an additional ~ ¼ sized AT rich region not located within the proximity of the subtelomeres, at position 7,011,437-7,026,387 bp (possible a neocentromere), of 14,951 bp length and an AT content of 80.9 %. Here we surveyed the functional annotation of neighbouring genes of the newly integrated centromere, neocentromere and subtelomeric sequences in *F. graminearum* (Results are given in Additional file 9). Although no overall pattern was found within 40 kb regions in proximity to the centromeres or the amino and carboxyl telomere sequences, two genes in common between these regions were found on the chromosome 4. These two genes (FGRRES_17709_M and FGRRES_08956_M) possess a NB-ARC signalling domain (PF00931) implicated in programmed cell death [46].

### Genes and other features associated with the proposed ancient chromosome fusion sites

In previous studies, *F. graminearum* was shown to contain only four chromosomes [4, 18], whilst in related Fusarium species a considerably higher numbers of chromosomes exist. For example, for *F. verticilloides* the presence of 11 chromosomes, and for *F. oxysporum*, 15 chromosomes were demonstrated using a whole-genome sequencing and comparative genomics approach [7]. Ma et al. [7] used the whole-genome alignment of *F. graminearum* and *F. verticilloides* to show that the 11 sequenced F. *verticilloides* chromosomes could be mapped to the four *F. graminearum* chromosomes and proposed that the smaller number of chromosomes in F. *graminearum* was the result of a series of ancient chromosome fusion events. The identified potential fusion points matched regions of high genomic diversity (Ma et al, Supplementary Fig. S3 [7]). Closer inspection of the centromeres in the RRes v4.0 reference sequence revealed repeat sequences of either MarCry-1_FO or LTR-Gypsy to embody a large proportion of the sequence content and also the AT rich regions within the subtelomeric loci. The fusion event proposed by Ma et al that led to *F. graminearum* centromeres were likely a result of fusion at these AT rich regions, which are found at various loci within each chromosome of *F. verticilloides*. This hypothesis is supported by repeating the alignment by Ma et al. but using the RRes v4.0 genomic reference sequence and focusing upon the centromere regions of *F. graminearum* that were not identified in the MIPS reference. Regions that aligned contained a number of annotated and non-annotated genes sequentially homologous in sequence and context. Different flanking sequences of AT rich regions of *F. verticilloides* from the same chromosomes align to flanks of the centromeres found in *F. graminearum* (see Additional file 10). The observation of different flanking sequences adjacent to AT rich regions from the same chromosome aligning to *F. graminearum* chromosome centromeres flanking sequences, suggests that fusing of multiple AT rich sequences to make the centromeres in *F. graminearum* did not include exclusively ancient centromere sequences either in part or full but incorporated other AT rich regions into the recombination event(s). This analysis provides further evidence in support of the existence of ancient chromosome fusion events in the *F. graminearum* genome which were not a direct result of the maintenance of ancient centromeres.

### Genes previously tested for a role in the disease causing ability of *F. graminearum*

The Pathogen-Host Interactions database (PHI-base) stores molecular and biological information on pathogen genes for which mutant phenotypes in pathogen-host interactions have been tested experimentally [47]. PHI-base currently provides information on > 2,800 pathogen genes, involving ~160 pathogenic species. There were *F. graminearum* 69 entries, including 59 putative transcription factors [48] and 8 kinases [49], an ATP citrate lyase ACL1 [50] and a syntaxin-like SNARE gene GzSYN1 [51] from PHI-base that had modified gene sequences in the RRes v4.0 gene set versus MIPS v3.2 (see Additional file 11). The kinases and transcription factor genes were analysed in high-throughput gene deletion studies for their virulence [48, 49]. Five of these genes were essential genes in *F. graminearum*, four genes when mutated caused reduced virulence, and one gene loss of pathogenicity. The remaining 59 genes were neither implicated in virulence nor essential for life. Due to changes in the gene model a re-evaluation of the function of each gene is recommended.

### Recombinant regions

Telomeres are established in many species as sites of high Single Nucleotide Polymorphism (SNP) frequency and structural rearrangement [52–54]. *F. graminearum* was shown to exhibit this trait but was also found to show interstitial regions of high diversity [4]. This was suggested to represent ancient telomeres as a consequence of chromosomal fusion of progenitor chromosomes. The mapping of markers used to establish these recombinant regions to the RRes v4.0 genome confirms these results with no observable changes in marker locations. The identification of the centromeres in the RRes genome and mapping of previously established markers shows that the high recombinant loci at the telomeres is on the long arm of the chromosomes, see Fig. 2. However, it is worth noting that markers are lacking for the carboxyl ends of the short arms of chromosome 1 and 3 due to new sequence inserted for the RRes v4.0 genome and may still show these regions to be highly recombinant in future studies. A comparison of total genes, secretome predicted genes, unique genes, and AT content by chromosomal arm reveals no pattern differences between areas of high or low recombination, (see Additional file 12). However it is clear that the secretome genes are predominantly found at areas of high recombination and within the subtelomeric regions, see Fig. 2.

### Repeat motifs and transposons

In the original BROAD analysis, the *F. graminearum* genome was found to contain very few transposon sequences annotated with gene ID’s and none were considered to be biologically active [4]. Active elements such as transposons are of interest because these are important contributors to genome evolution, including the creation of novel host genes and disruption of existing genes. The gene annotations identified with transposon protein domains are shown in Additional file 13. Of particular note was the new gene annotation, FGRRES_20409 is a gypsy-type long-terminal-repeat (LTR) retrotransposon, not previously included in the MIPS v3.2 assembly and the only retroviral transposon gene annotation found. RNA-seq supporting expression data in both the mycelium and spore was found suggesting this transposon is actively transcribed. However it was not found to have been included within any gene annotation leading to a truncation of the protein coding sequence. The sequence has been previously reported [GenBank:XM_389127] as a hypothetical protein partial mRNA in another PH-1 sequencing and annotation project but appears to have been an automated process with no reference in print. A comparison of centromere and subtelomeric AT rich regions repeat content shows a LTR gypsy sequence or MarCry-1_FO represented in each of these regions (see Additional file 14).

This study confirms previous findings in other species demonstrating the presence of transposon sequence in the centromeres [55]. The potential neocentromere identified in chromosome 3 shows a higher proportion of transposon content to the chromosome 3 centromere of 62.5 and 53 % respectively. In contrast to the subtelomeric regions there is a general trend towards a lower transposon content which further supports the hypothesis of a neocentromere at this location.

### BLAST comparisons

The original BLASTP analysis of the BROAD *F. graminearum* against *F. asiaticum, F. boothii, F. culmorum,* and *F. pseudograminearum* identified 704 species specific genes [4]. A similar BLASTP analysis (evalue 0.1) using high quality Fusarium genome sequences found on Ensembl fungi including: *F. culmorum*, *F. fujikuroi*, *F. oxysporum*, *F. verticilloides*, and *Nectria haematococca* using the RRes v4.0 genome annotations identified 741 species specific genes (see Additional file 15). Visualisation of these species specific genes loci shows no observable clustering (see Additional file 16).

BLASTP against the Fusarium cereal infecting subset, *F. culmorum, F. verticillioides*, and *F. fujikuroi* identified 769 unique genes. A conservative evalue of 0.1 for excluding proteins as part of the clade was used as little difference was observed as this value decreased. Comparison of the Fusarium and cereal clade species specific genes within the predicted RRes v4.0 secretome identified six genes that were *F. graminearum* unique and contain signal peptide sequences. Translated BLASTN of these six secreted *F. graminearum* unique sequences were explored against all Fusarium taxon entries in NCBI 12/12/2014 to test if the identifications were a result of missing or incorrect gene annotations. This analysis revealed one, FGRRES_20407, to have a hit below evalue 0.1 in both *Nectria haematococca* and *F. fujikuroi* and therefore this sequence is not unique to *F. graminearum*. The remaining five secreted sequences identified as unique to *F. graminearum* are; FGRRES_02309, FGRRES_15048, FGRRES_12652, FGRRES_15251, and FGRRES_20027.

The *F. graminearum* isolate genomes CS3005 and CS3096 are incomplete. This somewhat hinders inter-strain comparative analysis. However, our analysis of *F. graminearum* isolates CS3005 and CS3096 has revealed a total of 263 and 473 proteins, respectively identified as unique compared to *F. graminearum* PH-1. However a combinational analysis using complete genome sequences to identify SNP/Indel polymorphisms, expression analysis differences and unique proteins between isolates and the RRes v4.0 reference is required to identify the underlying reasoning behind these isolates genomic and phenotypic differences.

The original BROAD analysis of the *F. graminearum* genome had identified very little evidence of gene duplication and had discovered an active RIP mechanism that had eliminated duplicated sequences. An integral protein of the RIP mechanism is the presence of a DNA methyltransferase RIP defective gene (RID) [Swiss-Prot: Q8NJW0], reported in *Neurospora crassa* [56]. BLASTP of the proteome of *F. graminearum* versus the *RID* gene from *N. crassa* reveals one protein, FGRRES_08648, of similar identity (380 aa alignment length of 42.0 %) but with an additional DNA methylase C-5 cytosine-specific active site. This protein is hypothesised to represent the *RID* gene in *F. graminearum* and further support the activity of RIP*.* The transposon class TcMar-Fot1, FGRRES_15950, FGRRES_17263, FGRRES_16975, FGRRES_13431_M are all single exon predictions but for FGRRES_13431_M which is multi-exon. A previous analysis by Cuomo et al. has shown RIP induced SNPs in this class of transposons however an alignment was not reported. A BLASTN alignment of the region of FGRRES_13431_M versus the other TcMar-Fot1 class of transposons reveals that the transposon has had two stop codons introduced which explains the multi-exon prediction from the gene software. FGRRES_16975 has also a stop codon introduced causing a truncation. In addition to the induced SNPs between these transposons we can report that this has led to stop codon introduction, likely terminating the functionality of the transposons discussed and additional evidence to the process of RIP in *F. graminearum*.

A reciprocal BLASTP of the *F. graminearum* proteome identified two duplicated protein sequences, one of which had 100 % nucleotide identity. The first is a Tigger transposon (FGRRES_15950 and FGRRES_17263) at position 6,157,128 chromosome 1 and position 5,484,124 chromosome 4 respectively which had identical nucleotides. The second is the histone H4 protein FGRRES_20411 (to be re-designated as FGRRES_04289 in RRes v4.1) and FGRRES_05491_M with 20 SNPs: C:G = 1, C:A = 1, T:G = 6, G:A = 12 (see Additional file 17). The sequence of FGRRES_05491_M is longer than FGRRES_20411 with an additional 62 aa at the amino end of the protein which includes a signal peptide of 20 aa which is predicted to target the protein to the mitochondria. Within the RRes v4.0 annotation there are two H3 proteins, one H1, one H1/H5, three H2 (A, B, C), but three H4 proteins of which two have the aforementioned 100 % matching sequences present.

In a further analysis, we explored gene families based upon protein sequence similarity which grouped sequences into no more than four using a similar methodology to Klenk et al. and Heidelberg et al. [57, 58]. The average family size is 2.09 and the largest 18 families identified included 6 families of amino acid transporters, three major facilitator families, three transposon families, three tyrosinases (pigment formation) families, one aldehyde/histidinol dehydrogenase family, one unknown function family and a family of nucleoside triphosphase hydrolases. The remaining families were no greater than 2. This suggests that few genes have formed via multi-duplications arising from a single progenitor gene to form these small multigene families.

## Discussion

The *de novo* genome assembly using *de Bruijn* assembler software, such as SOAPdenovo2 [59], requires the use of a k-mer value which requires a set sequence length in order to align and join sequenced reads but this is limited to the length of the reads i.e. 100 bp Illumina reads. As the k-mer value rises, this increases the specificity but reduces sensitivity. Therefore contigs of a repetitive nature such as AT rich regions within centromeres, have a higher chance of assembling into a single sequence as the k-mer value increases. There is a limitation to this approach, because as the k-mer value is increased greater sequencing coverage is needed to compensate for the reduced sensitivity. Using 100 bp Illumina reads and a ~85 x genome coverage, a k-mer value of approximately 61 resulted in the highest N50 assembly. The presence of AT rich regions or repetitive transposons loci are likely to cause breaks in the resulting contigs [60]. Using a greater k-mer value can in some cases create longer sequence contigs for these loci and potentially result in contigs that span these repetitive regions along with the non-repetitive flanks. However combining such assemblies can be manually time consuming for genomes with many repetitive loci and genomes larger than 36 Mbp. The multi k-mer approaches like those that are available to SOAPdenovo2 can combine these two scenarios to produce a superior assembly with little manual curation. We were successful in using this approach to fully assemble the *F. graminearum* genome. For genomes of a similar size but contain more repetitive sequences, this strategy might be less successful but is worthwhile investigating. For *F. graminearum* the multi-kmer approach resulted in the insertion of previously unknown sequences, identification of a significant mis-assembly in chromosome 1 at the amino end of the MIPS v3.2 genome, extending several internal AT rich regions, placing 12 supercontigs within chromosomes, and for the first time identifying for each of the four chromosomes the telomeric sequence (TAACCC) and centromeric DNA.

The analysis of centromeric DNA in filamentous fungi is difficult due to the fact that centromeric regions are comprised of heterogenous, repetitive, AT-rich sequences [61]. Identification of the size and position of the centromeres on each of the four *F. graminearum* chromosomes permitted an examination of the annotations within the immediate vicinity versus annotation within the rest of the chromosome arms. However comparison of total gene content, the secretome gene subset, *F. graminearum* unique genes, and GC content revealed no obvious patterns (see Additional file 18). In *Saccharomyces cerevisiae* short point centromeres of 120 bp exist [62]. In contrast in many other fungi including *Schizosaccharomyces pombe*, the human pathogenic fungus *Candida albicans* and in the filamentous fungus *N. crassa* regional centromeres of considerable length are prevalent [63]. In *C. albicans,* regional centromeres are composed of unique 3-4.5 kb sequences [64]. In the case of *N. crassa*, centromeres are composed of degenerate transposons, retrotransposons and simple sequence repeats, with no gene sequences. Our BLAST analysis (e < 0.00001) confirmed the absence of genes or distinctive DNA elements for the four centromeric regions in *F. graminearum.* The centromeric composition, despite its AT rich >90 %, was sufficiently varied to assemble using the four re-sequencing data sets we had available with an insert distance of between 408 and 449 and the use of k-mer values >81, see Additional file 19. The average size of the four centromeres was found to be 59,771 bp, and for chromosome 3 our analysis revealed the existence of an additional smaller AT-rich region of 15 kb. This region is approximately ¼ the size of the designated centromeric region and may comprise a neocentromeric region or the remainder of centromere repositioning event. The genes localised immediately adjacent to the four centromeres did not include genes implicated in virulence. Several sequences with predicted functions in programmed cell death and chromosome segregation, replication and chromatin remodelling were found to be located in close proximity to the centromeres. We propose that the availability of centromeric sequence information for *F. graminearum* may be exploited in several ways. (1) Although no evidence exists, they may help construct artificial chromosomes. Currently none are available for filamentous fungi. (2) Assist in cancer research. In several types of human cancers, centromeres were found to be deleted and kinetochores instead assemble on neocentromeres. Experiments in both *S. pombe* and *C. albicans* were successful in deleting centromeric regions and identifying neocentromeres. (3) To identify fungal specific drug targets. Kinetochore proteins binding to the centromeric regions in filamentous fungi during mitosis were proposed to be highly fungal-specific and provide pertinent specific drug targets [61]. Binding studies between fungal specific proteins and centromeric DNA regions may reveal suitable small-molecule drugs.

The annotation of the genome was comprehensive. First we took the BROAD (FG3) genome and the gene models provided by MIPS (v3.2). Then we included additional annotation from a range of different sources to provide evidence for new gene models and to modify existing gene models. This additional annotation evidence was obtained by using RNA-seq data from the mycelium and spores of strain PH-1 [EMBL:SRP039087] as well as the mycelium of strain Z-3639 harvested 24 h after inoculation of wild-type conidia in complete medium RNA-seq [EMBL:SAMN02400310]. In addition Fusarium protein data was taken from Uniprot and we also used the extensive EST libraries generated by Sanger sequencing available from the Cogeme EST transcripts database [27]. As a consequence, a total 412 new gene models were predicted and 1529 gene models have been modified.

The new 412 gene predictions and curated gene models are a significant step towards a fully annotated genome. However further manual curation is required by the community using RNA-seq supporting evidence from multiple biological situations to achieve the high level of novel transcriptome information needed to complete this important activity. Currently, this process is organised using the public Ensembl Fungi websites [22] and the phytopathogenic specific website, PhytoPathdb [23]. Custom tracks can be displayed against the completed *F. graminearum* alongside any new gene model annotations and all changes are shared via a mailing list. Group members can contribute, view and discuss updated gene models prior to being incorporated into future Ensembl Fungi versions (see Additional file 20 for instructions).

The study of the *F. graminearum* gene repertoire predicted to code for secreted proteins is highly significant to research aimed at understanding the pathogenic process due to the their hypothesised roles in penetration, host tissue necrosis and host immune subversion [65]. The rapid rearrangement in these regions may promote rapid evolution of secretory genes in response to host responses leading to species specific genes and virulence hot spots. For example, in the *Aspergillus* species, species unique secondary metabolite genes are enriched in subtelomeric regions [66, 67]. The only similarly secreted and *F. graminearum* unique protein that has a cysteine content of above 5 % (9 %) is FGRRES_15251 and is located in the subtelomeric region of chromosome 2, at 8,527,286 bp. These characteristics are a hallmark of an effector protein in other filamentous pathogenic species and of potential interest to gene deletion studies. The reconfirmation of the identification of secretory genes and the identification of new secretory genes within these subtelomeric and the high recombinant regions previously demonstrated by Ma et al. and Brown et al. supports this hypothesis. The identification of a predominance of the tRNA annotations within the genes predicted to code for secreted proteins, not previously noted, may be hypothesised to represent an evolutionary bias for improved response to stimulus such as host detection resulting in a rapid response to infection and an overall switch to the pathogenic process.

The detailed analysis of the newly predicted *F. graminearum* secretome, revealed two new secretory and one non-secretory proteins found in the RRes v4.0 predictions of potential importance to virulence and also serves to illustrate the significance of the complete genome and high quality annotations to the conclusions of research findings going forward. FGRRES_20327 is annotated as secreted and possessing alpha-N-arabinofuranosidase activity and is located in a new coding sequence island approximately 2 kb within two AT rich regions of approximately 9 kb length at the carboxyl end of chromosome 3. Whilst FGRRES_20176 is annotated as a secreted pathogenicity protein with transporter activity. Both genes were previously only identified in the NGS genome study of the high virulence *F. graminearum* Australia isolate CS3005 (FG05_30019 and FG05_35058, respectively). A third example, is the new cysteine-rich potential virulence gene (FGRRES_17235_M) previously only reported in the highly virulent strain CS3005 of *F. graminearum* which contains several features characteristic of many apoplastic fungal effectors shown to inhibit plant chitinases used to prevent pathogen associated molecular pattern triggered immunity thereby preventing induction of host defences. The previous identification of these proteins in CS3005 but not PH-1 was therefore the result of the incompleteness of the PH-1 genome.

The reanalysis of the well-studied *Tri* and zearalenone gene clusters proved to be interesting. A previous study had observed the co-expression of three flanking genes, namely 03531 (OrfA), 03530 (OrfB) and 3529 with the core *Tri* genes and extended the list of genes involved in trichothecene DON production and maintenance [68]. In the current study, one gene was modified in the *Tri* gene cluster and in the zearalenone gene clusters. However with the publically available RNA-seq data and manual curation of other newly identified genes, then it may be possible to identify and associate other sequences to these two key pathways and iteratively improve their curation and annotation. These results highlight the need for re-appraisal of existing functional data to inform the current protein function studies on these two important toxin pathways.

The absence of genes that arose from duplications in *F. graminearum* reflects the activity of a process found in many fungi [69] namely, repeat-induced point mutation (RIP) in which mutations are caused in duplicated sequences [55]. This process is hypothesised to be a defence mechanism against transposable elements which are common to viral/bacterial infection. A previous study has shown the introduction of C:G to T:A transition mutations during the sexual cycle in *F. graminearum* transposons [4]. The identification of two histone H4 proteins of identical protein content but different DNA sequences suggests these two sequences may be the consequence of a recent duplication event which is in the process of RIP. However the introns of the proteins are substantially different and SNP C:G or T:A transitions are not predominant in the shared coding regions. This suggests that these H4 proteins containing identical coding sequences but for an additional sequence including a signal peptide that is predicted to target the mitochondria for FGRRES_05491_M, may not be in the process of RIP but is present due to important functional reasons currently unknown.

The large repeating rRNA containing sequence at the carboxyl end of chromosome 4 is similarly found in the human genome on chromosome 21 with adjacent unknown bases. It is highly likely to exist in other genome reference sequences at the end of a chromosome interspersed with N bases and/or as an unplaced contigs. By manual investigation such features where found in the genome references sequences (Ensembl Fungi v23) of *F. fujikuroi* and *F. oxysporum* f. sp. *lycopersici* at the respective amino and carboxyl ends of chromosome 2. In *F. solani* such sequences were found as unscaffolded contigs which are likely to originate from the amino or carboxyl end of a chromosome sequence yet unplaced. This feature may be common among the Fusarium clade and beyond but requires a greater number of genomic reference sequences to be completed before this feature can be thoroughly explored.

The genome and annotation sequences have been deposited into ENA and displayed at Ensembl fungi and with the dedicated plant pathogen PhytoPathdb database to permit public access to the data and tracking of versions. Information can be linked to the genome from other databases, such as the Pathogen-Host Interaction entries [47] to efficiently access phenotypic information from pathogenicity testing involving single and multiple gene deletion isolates generated mostly in the reference strain PH-1. The presentation in Ensembl fungi/PhytoPathdb will aid comparative genomics studies by the identification of homologous and orthologous genes with other representative fungal genomes which are continually increasing and being updated.

## Conclusion

Using whole shotgun re-sequencing of *F. graminearum* we have comprehensively completed the genomic sequence by replacing unknown bases, placing supercontigs within their correct loci, correcting assembly errors, and inserting new sequences which include for the first time complete AT rich sequences such as centromere sequences and subtelomeric regions. The identification of a retroviral transposon on chromosome 4 shows a potential route that *F. graminearum* is continuing to evolve in particular to environmental stresses, such as fungicides, and diverge from related fungal species.

An analysis of 412 new gene models and 1529 curated gene models reveals 7 new and 25 modified transcription regulator protein predictions, three new targets for virulence mechanisms, and includes a range of proteins that may require further re-testing as demonstrated by the 59 entries with modified sequences reported in PHI-base which are reported as not being virulently associated. All these changes will greatly improve the analysis of gene function and the reinterpretation of the considerable number of transcriptomics data sets deposited by the international Fusarium community which are available at PLEXdb [70]. The redefined subset of genes predicted to comprise the refined secretome, verifies the previously recognised secretome predominantly within previously identified hotspot location for this gene type in the genome, namely the subtelomeric and highly recombinatorial regions. The *F. graminearum* genome can now be comprehensively studied with the knowledge that a full set of gene models and surrounding genomic sequence can be probed in combination with non-coding RNA annotation to identify pathogenicity mechanisms.

In summary, we present a fully completed *F. graminearum* isolate PH-1 genome and manually curated annotation using RNA-seq evidence which provides the optimum resource to perform interspecies comparative analyses and gene function studies using various reverse genetics approaches. The availability of the RRes v4.0 genome should also permit in the near future a detailed inter-comparison of historic and modern *F. graminearum* isolates collected from diverse geographical locations, plant species as well as fungicide application and crop rotation regimes.

### Availability of data and materials

Sequence Read Archive (SRA) accession numbers for the four cells of Illumina sequencing are [EMBL:ERS430784, EMBL:ERS430785, EMBL:ERS430786, EMBL:ERS430787]. The RRes genomic and mitochondrial sequence for the *F. graminearum* strain PH-1 is available at the European Nucleotide Archive [EMBL:HG970330, EMBL:HG970331, EMBL:HG970332, EMBL:HG970333, EMBL:HG970334, EMBL:HG970335]. An OmniMap instance of the RRes v4.0 FG genome which includes files to reproduce images contained herein is available for download [71].

## Methods

### Fungal growth, DNA preparation and sequencing

The fungal strain PH-1 (ATCC MYA-4620/FGSC 9075/NRRL 31084) was cultured as described [72]. Genomic fungal DNA for sequencing was extracted using the CTAB protocol [73] and purified using a Qiagen Kit (Qiagen Ltd, Crawley, West Sussex, UK). High-quality genomic DNA was then submitted to the genome analysis centre (TGAC, Norwich, UK) for generation of a 0.8-kb fragment library. The Illumina HiSeq 2000 sequencing platform (San Diego, CA) was used to produce 100-bp paired-end reads [74].

### Assembly and alignment

No pre-processing of reads took place. The software Novoalign3 (version 3.01.02) and BWA (version 0.7.5a-r405) were used to align the sequencing reads, with default parameters, to the *F. graminearum* reference sequence (MIPS version FG3.2 [75]). Alignments were converted from the sequence alignment map (SAM) format to binary alignment map (BAM), and the BAM files were sorted and indexed using SAMtools (version 0.1.19). Visualisations were done using Tablet [76] (version 1.13.07.31).

For *de novo* assembly, the software SOAPdenovo2 (version 2.0.4) was used with different k-mer values: 61, 63, 71, 81, and 91. The resulting assemblies were used to correct and extend MIPS version FG3.2 sequence and orientation where possible. Reference sequence statistics were extracted from Tablet and Geneious (version 6.1 created by Biomatters). LASTZ (version 1.02.00) was used from within Geneious to align genomic sequences.

### Genome annotation

The assembled genome was annotated using the MAKER (version 2.30) [21] annotation pipeline with RepeatMasker (version 4.0.5) [77]. A *F. graminearum* specific repeat library was constructed using RepeatModeler (version 1.0.7) and supplied to MAKER for the repeat masking step. Gene calls were generated using FGENESH (version 3.1.2) [78] using the Fusarium matrix, AUGUSTUS (version 2.7) [79] using *F. graminearum* species model, GeneMark [80] and SNAP [81], which was trained using all the Fusarium proteins in UNIPROT with the keyword “Fusarium” which had evidence, ESTs from Cogeme [28], and trinity assemblies using RNA-seq from the mycelium and spores of PH-1, and wild type Z-3639. This evidence was provided to MAKER as hints to the annotation. The final annotation set produced by MAKER and manually curated to include the MIPS v3.2 annotation is summarized in Table 9. Non-coding RNA were identified using default settings with both tRNAscan-SE-1.3.1 [82] and Infernal-1.1 [83]. InterProscan-5.7-48.0 (analyses: TIGRFAM-13.0, ProDom-2006.1, SMART-6.2, HAMAP-201311.27, SignalP-EUK-4.0, PrositePAtterns-20.97, PRINTS-42.0, SuperFamily-1.75, Panther-9.0, Gene3d-3.5.0, SignalP-GRAM_POSITIVE-4.0, PIRSF-2.84, SignalP-GRAM_NEGATIVE-4.0, PfamA-27.0, PrositeProfiles-20.97, Phobius-1.01, TMHMM-2.0c, Coils-2.2) was used to determine whether to correct a gene model based upon new or extended protein domains.

### Gene statistics, interproscan domain, GO and enzyme comparisons

Blast2GO V.2.7.2 was used with Decypher BLASTP search’s with an E-value of 0.001 against the NCBI nr database from 04/09/14 and 15/09/14 NCBI nr database for the 412 new gene and modified protein sets respectively, filtered using Blast2GO annotation algorithm with settings, E-value filter 0.000001, Annotation CutOff 55, GO weight 5, Hsp-Hit Coverage CutOff 0, and GO and enzyme code annotated using a local GO database from 07/2014 with 41,436 GOs available and 4,098 Enzymes available. Interproscan results were imported in to Blast2GO and the GO annotations merged. Annotation statistics were produced using Eval V.2.2.8 and Geneious.

### BLAST comparisons

Unique proteins from the annotations of RRes V4.0, and the *F. graminearum* isolates CS3005 and CS3096 were assigned using BLASTP with an evalue 0.1 using either: 1) Fusarium subset which included; F*. culmorum, F. fujikuroi, F. oxysprum f.sp. lycopersici (4287), F. verticilloides, Gibberella moniliformis, Nectria haematococca*, or 2) Fusarium cereal infecting subset including: *F. culmorum, F. verticillioides, F. fujikuroi.*

### Secretome identification

InterProscan-5.7-48.0 was used to identify signal and transmembrane domains. Proteins smaller than 20 amino acids were excluded. ProtComp (Version 9.0) [84] and result columns, LocDB and PotLocDB were used to exclude GPI anchored membrane proteins and other non-extracellular loci proteins. WoLfPSort [85] (extracellular score > 17, WoLfPSort) was used to identify final destination and big-PI [86] to further remove GPI-anchored proteins.

### SNP calling

BWA-MEM [87] was used to align the sequencing reads to the BROAD *F. graminearum* (FG3) reference using default parameters, followed by conversion to BAM format and sorting by coordinate using SAMtools (v0.1.19-44428 cd) [88]. SNPs and indels were called using SAMtools mpileup followed by VarScan (v2.3.7) using default settings.

## Additional files

Additional file 1:
**Figures showing H3K9me3 binding in the MIPS reference with alignments of RRes and MIPs reference to highlight where centromeric sequence was present in the Connolly et al. study.**
Additional file 2:
**A table listing the gene models in RRes v4.0 deleted from the MIPS annotation.**
Additional file 3:
**A table of RRes v4.0 and MIPS v3.2 binned protein lengths.**
Additional file 4:
**A table of RRes v4.0 and MIPS exons per transcript.**
Additional file 5:
**A table listing the BLAST and GO annotations of the RRes v4.0 new gene prediction subset.**
Additional file 6:
**A description and table listing the RRes secretome gene ID’s and those unique to the earlier Brown et al. [** 42 **]** ***vs.*** **RRes v4.0 secretome.**
Additional file 7:
**A figure of RRes v4.0 secretome set and RRes and Brown et al. unique secretome gene prediction comparison.**
Additional file 8:
**A table of Gene ID’s with GO term 0001071 which are identified as having nucleic acid binding transcription factor activity from either altered gene predictions or genes that were not found in the MIPS v3.2 annotation.**
Additional file 9:
**A table of distal centromere, possible neocentromere and telomere gene ID’s and annotations.**
Additional file 10:
**A table of the alignment positions of flanking sequences of AT rich regions from the** ***F. verticillioides*** **chromosomes to the sequences flanking the centromeres in RRes v4.0** ***F. graminearum*** **.**
Additional file 11:
**A table of RRes v4.0 gene models that have changed since MIPS v3.2 that are present in PHI-base.**
Additional file 12:
**A table of RRes v4.0** ***F. graminearum*** **recombinant and non-recombinant region comparison.**
Additional file 13:
**A description of RRes repeat masking details and a table of gene ID’s identified with transposon domains.**
Additional file 14:
**A table of transposon and repeat content of centromeres and subtelomeric AT rich regions.**
Additional file 15:
**A table of** ***Fusarium graminaerum*** **species specific genes identified in the RRes v4.0 gene annotation.**
Additional file 16:
**A figure representing chromosomal locations of** ***Fusarium graminaerum*** **species specific gene ID’s identified in the RRes v4.0 gene annotation.**
Additional file 17:
**A figure of the nucleotide alignment of genomic sequence of duplicate containing H4 protein sequences FGRRES_20411 (FGSG_04289) and FGRRES_05491_M.**
Additional file 18:
**A table of RRes v4.0 all genes, secretome subset genes, and unique gene sets per chromosome arm.**
Additional file 19:
**Shows the mapped read coverage across the centromeres and the corresponding GC content.**
Additional file 20:
**A set of instructions for how to stay updated and contribute to** ***F. graminearum*** **PH-1 gene models changes prior to future Ensembl Fungi version releases.**

## References

[1] Swain MT, Tsai IJ, Assefa SA, Newbold C, Berriman M, Otto TD. A post-assembly genome-improvement toolkit (PAGIT) to obtain annotated genomes from contigs. Nat Protoc. 2012;7(7):1260–84. 10.1038/nprot.2012.068 PMC3648784 22678431

[2] Gross SS, Do CB, Sirota M, Batzoglou S. CONTRAST: a discriminative, phylogeny-free approach to multiple informant de novo gene prediction. Genome Biol. 2007;8(12):R269. 10.1186/gb-2007-8-12-r269 PMC2246271 18096039

[3] Srivastava SK, Huang X, Brar HK, Fakhoury AM, Bluhm BH, Bhattacharyya MK. The genome sequence of the fungal pathogen Fusarium virguliforme that causes sudden death syndrome in soybean. PLoS One. 2014;9(1):e81832. 10.1371/journal.pone.0081832 PMC3891557 24454689

[4] Cuomo CA, Guldener U, Xu JR, Trail F, Turgeon BG, Di Pietro A, et al. The Fusarium graminearum genome reveals a link between localized polymorphism and pathogen specialization. Science. 2007;317(5843):1400–2. 10.1126/science.1143708 17823352

[5] BROAD INSTITUTE. Fungal Genomics. 2015. http://www.broadinstitute.org/scientific-community/science/projects/fungal-genome-initiative/fungal-genome-initiative. Accessed 01 July 2014.

[6] Jeong H, Lee S, Choi GJ, Lee T, Yun SH. Draft genome sequence of Fusarium fujikuroi B14, the causal agent of the Bakanae disease of rice. Genome Announc. 2013. 1(1):e00035–00013 10.1128/genomeA.00035-13 PMC3587928 23472226

[7] Ma LJ, van der Does HC, Borkovich KA, Coleman JJ, Daboussi MJ, Di Pietro A, et al. Comparative genomics reveals mobile pathogenicity chromosomes in Fusarium. Nature. 2010;464(7287):367–73. 10.1038/nature08850 PMC3048781 20237561

[8] Moolhuijzen PM, Manners JM, Wilcox SA, Bellgard MI, Gardiner DM. Genome sequences of six wheat-infecting fusarium species isolates. Genome Announc. 2013. 1(5):e00670-13-e00670-13 10.1128/genomeA.00670-13 PMC3764410 24009115

[9] Gardiner DM, Stiller J, Kazan K. Genome Sequence of Fusarium graminearum Isolate CS3005. Genome Announc. 2014. 2(2):e00227-14 10.1128/genomeA.00227-14 PMC3990742 24744326

[10] Jiang D, Zhu W, Wang Y, Sun C, Zhang KQ, Yang J. Molecular tools for functional genomics in filamentous fungi: recent advances and new strategies. Biotechnol Adv. 2013;31(8):1562–74. 10.1016/j.biotechadv.2013.08.005 23988676

[11] Goswami RS, Kistler HC. Heading for disaster: Fusarium graminearum on cereal crops. Mol Plant Pathol. 2004;5(6):515–25. 10.1111/j.1364-3703.2004.00252.x 20565626

[12] Lee T, Oh DW, Kim HS, Lee J, Kim YH, Yun SH, et al. Identification of deoxynivalenol- and nivalenol-producing chemotypes of Gibberella zeae by using PCR. Appl Environ Microbiol. 2001;67(7):2966–72. 10.1128/AEM.67.7.2966-2972.2001 PMC92968 11425709

[13] Lysoe E, Klemsdal SS, Bone KR, Frandsen RJ, Johansen T, Thrane U, et al. The PKS4 gene of Fusarium graminearum is essential for zearalenone production. Appl Environ Microbiol. 2006;72(6):3924–32. 10.1128/AEM.00963-05 PMC1489647 16751498

[14] Desjardins AE, Hohn TM, McCormick SP. Trichothecene biosynthesis in Fusarium species: chemistry, genetics, and significance. Microbiol Rev. 1993;57(3):595–604. 10.1128/mr.57.3.595-604.1993 PMC372927 8246841

[15] Proctor RH, Hohn TM, McCormick SP. Reduced virulence of Gibberella zeae caused by disruption of a trichothecene toxin biosynthetic gene. Mol Plant Microbe Interact. 1995;8(4):593–601. 10.1094/mpmi-8-0593 8589414

[16] Wu F, Guclu H. Aflatoxin regulations in a network of global maize trade. PLoS One. 2012;7(9):e45151. 10.1371/journal.pone.0045151 PMC3458029 23049773

[17] Urban M, Hammond-Kosack K. Molecular genetics and genomic approaches to explore Fusarium infection of wheat floral tissue. In: Fusarium Genomics and Molecular and Cellular Biology (Proctor, R.H. and Brown, D., eds), Chapter 12 Norwich, Norfolk, UK: Horizon Scientific Press; 2013. p. 43–79.

[18] Gale LR, Bryant JD, Calvo S, Giese H, Katan T, O'Donnell K, et al. Chromosome complement of the fungal plant pathogen Fusarium graminearum based on genetic and physical mapping and cytological observations. Genetics. 2005;171(3):985–1001. 10.1534/genetics.105.044842 PMC1456848 16079234

[19] Wong P, Walter M, Lee W, Mannhaupt G, Munsterkotter M, Mewes HW, et al. FGDB: revisiting the genome annotation of the plant pathogen Fusarium graminearum. Nucleic Acids Res. 2011;39(Database issue):D637–9. 10.1093/nar/gkq1016 PMC3013644 21051345

[20] Güldener U, Mannhaupt G, Munsterkotter M, Haase D, Oesterheld M, Stumpflen V, et al. FGDB: a comprehensive fungal genome resource on the plant pathogen Fusarium graminearum. Nucleic Acids Res. 2006;34(Database issue):D456–8. 10.1093/nar/gkj026 PMC1347389 16381910

[21] Holt C, Yandell M. MAKER2: an annotation pipeline and genome-database management tool for second-generation genome projects. BMC Bioinformatics. 2011;12:491. 10.1186/1471-2105-12-491 PMC3280279 22192575

[22] Ensembl Fungi. 2015. http://fungi.ensembl.org/. Accessed 24 Jan 2015.

[23] PytoPath. 2015. http://www.phytopathdb.org/. Accessed 24 Jan 2015.

[24] Blackburn EH. Structure and function of telomeres. Nature. 1991;350(6319):569–73. 10.1038/350569a0 1708110

[25] Meyne J, Ratliff RL, Moyzis RK. Conservation of the human telomere sequence (TTAGGG)n among vertebrates. Proc Natl Acad Sci U S A. 1989;86(18):7049–53. 10.1073/pnas.86.18.7049 PMC297991 2780561

[26] Huson DH, Auch AF, Qi J, Schuster SC. MEGAN analysis of metagenomic data. Genome Res. 2007;17(3):377–86. 10.1101/gr.5969107 PMC1800929 17255551

[27] Connolly LR, Smith KM, Freitag M, The Fusarium graminearum histone H3 K27 methyltransferase KMT6 regulates development and expression of secondary metabolite gene clusters. PLoS Genetics, 2013. 9(10):e1003916 10.1371/journal.pgen.1003916 PMC3814326 24204317

[28] Soanes DM, Skinner W, Keon J, Hargreaves J, Talbot NJ. Genomics of phytopathogenic fungi and the development of bioinformatic resources. Mol Plant Microbe Interact. 2002;15(5):421–7. 10.1094/MPMI.2002.15.5.421 12036272

[29] Dixon DC, Cutt JR, Klessig DF. Differential targeting of the tobacco PR-1 pathogenesis-related proteins to the extracellular space and vacuoles of crystal idioblasts. EMBO J. 1991;10(6):1317–24. 10.1002/j.1460-2075.1991.tb07650.x PMC452789 2026137

[30] Shen K, Wang Y, Hwang Fu YH, Zhang Q, Feigon J, Shan SO. Molecular mechanism of GTPase activation at the signal recognition particle (SRP) RNA distal end. J Biol Chem. 2013;288(51):36385–97. 10.1074/jbc.M113.513614 PMC3868752 24151069

[31] Bovia F, Strub K. The signal recognition particle and related small cytoplasmic ribonucleoprotein particles. J Cell Sci. 1996;109(Pt 11):2601–8. 10.1242/jcs.109.11.2601 8937977

[32] Dunin-Horkawicz S, Feder M, Bujnicki JM. Phylogenomic analysis of the GIY-YIG nuclease superfamily. BMC Genomics. 2006;7:98. 10.1186/1471-2164-7-98 PMC1564403 16646971

[33] Proctor RH, McCormick SP, Alexander NJ, Desjardins AE. Evidence that a secondary metabolic biosynthetic gene cluster has grown by gene relocation during evolution of the filamentous fungus Fusarium. Mol Microbiol. 2009;74(5):1128–42. 10.1111/j.1365-2958.2009.06927.x 19843228

[34] Hallen-Adams HE, Wenner N, Kuldau GA, Trail F. Deoxynivalenol biosynthesis-related gene expression during wheat kernel colonization by Fusarium graminearum. Phytopathology. 2011;101(9):1091–6. 10.1094/PHYTO-01-11-0023 21521001

[35] Pestka JJ,Smolinski AT. Deoxynivalenol: toxicology and potential effects on humans. J Toxicol Environ Health B Crit Rev. 2005;8(1):39–69. 10.1080/10937400590889458 15762554

[36] Urban M, Willighagen E, Chichester C, Kutmon M. WikiPathways. DON mycotoxin biosynthesis (Gibberella zeae). 2014. http://www.wikipathways.org/index.php/Pathway:WP2258. Accessed 01 Jul 2014.

[37] Kim YT, Lee YR, Jin J, Han KH, Kim H, Kim JC, et al. Two different polyketide synthase genes are required for synthesis of zearalenone in Gibberella zeae. Mol Microbiol. 2005;58(4):1102–13. 10.1111/j.1365-2958.2005.04884.x 16262793

[38] Gardiner DM, Kazan K, Manners JM. Novel genes of Fusarium graminearum that negatively regulate deoxynivalenol production and virulence. Mol Plant Microbe Interact. 2009;22(12):1588–600. 10.1094/MPMI-22-12-1588 19888824

[39] McCormick SP, Harris LJ, Alexander NJ, Ouellet T, Saparno A, Allard S, et al. Tri1 in Fusarium graminearum encodes a P450 oxygenase. Appl Environ Microbiol. 2004;70(4):2044–51. 10.1128/AEM.70.4.2044-2051.2004 PMC383062 15066795

[40] Brown NA, Hammond-Kosack KE. Secreted biomolecules in fungal plant pathogenesis, in fungal biomolecules: sources, applications and recent developments, V. K. Gupta, S. Sreenivasaprasad, and Robert L. Mach, Editor. John Wiley & Sons, Ltd, Chichester, UK; 2015.

[41] Molloy S. Fungal physiology. Ustilago takes control. Nat Rev Microbiol. 2011;9(12):832–3. 10.1038/nrmicro2705 22085849

[42] Brown NA, Antoniw J, Hammond-Kosack KE. The predicted secretome of the plant pathogenic fungus Fusarium graminearum: a refined comparative analysis. PLoS One. 2012;7(4):e33731. 10.1371/journal.pone.0033731 PMC3320895 22493673

[43] Yang F, Jensen JD, Svensson B, Jorgensen HJ, Collinge DB, Finnie C. Secretomics identifies Fusarium graminearum proteins involved in the interaction with barley and wheat. Mol Plant Pathol. 2012;13(5):445–53. 10.1111/j.1364-3703.2011.00759.x PMC6638632 22044785

[44] Paper JM, Scott-Craig JS, Adhikari ND, Cuomo CA, Walton JD. Comparative proteomics of extracellular proteins in vitro and in planta from the pathogenic fungus Fusarium graminearum. Proteomics. 2007;7(17):3171–83. 10.1002/pmic.200700184 17676664

[45] Villasante A, Mendez-Lago M, Abad JP, Montejo de Garcini E. The birth of the centromere. Cell Cycle. 2007;6(23):2872–6. 10.4161/cc.6.23.5047 18156801

[46] van der Biezen EA, Jones JD. The NB-ARC domain: a novel signalling motif shared by plant resistance gene products and regulators of cell death in animals. Curr Biol. 1998;8(7):R226–7. 10.1016/s0960-9822(98)70145-9 9545207

[47] Urban M, Pant R, Raghunath A, Irvine AG, Pedro H, Hammond-Kosack KE. The Pathogen-Host Interactions database (PHI-base): additions and future developments. Nucleic Acids Res. 2015;43(Database issue):D645–55. 10.1093/nar/gku1165 PMC4383963 25414340

[48] Son H, Seo YS, Min K, Park AR, Lee J, Jin JM, et al. A phenome-based functional analysis of transcription factors in the cereal head blight fungus, Fusarium graminearum. PLoS Pathog. 2011;7(10):e1002310. 10.1371/journal.ppat.1002310 PMC3197617 22028654

[49] Wang C, Zhang S, Hou R, Zhao Z, Zheng Q, Xu Q, et al. Functional analysis of the kinome of the wheat scab fungus Fusarium graminearum. PLoS Pathog. 2011;7(12):e1002460. 10.1371/journal.ppat.1002460 PMC3245316 22216007

[50] Son H, Lee J, Park AR, Lee YW. ATP citrate lyase is required for normal sexual and asxexual development in Gibberella zeae. Fungal Genet Biol. 2011;48(4):408–17. 10.1016/j.fgb.2011.01.002 21237280

[51] Hong SY, So J, Lee J, Min K, Son H, Park C, et al. Functional analyses of two syntaxin-like SNARE genes, GzSYN1 and GzSYN2, in the ascomycete Gibberella zeae. Fungal Genet Biol. 2010;47(4):364–72. 10.1016/j.fgb.2010.01.005 20102747

[52] Mefford HC, Trask BJ. The complex structure and dynamic evolution of human subtelomeres. Nat Rev Genet. 2002;3(2):91–102. 10.1038/nrg727 11836503

[53] Gardner MJ, Hall N, Fung E, White O, Berriman M, Hyman RW, et al. Genome sequence of the human malaria parasite Plasmodium falciparum. Nature. 2002;419(6906):498–511. 10.1038/nature01097 PMC3836256 12368864

[54] Winzeler EA, Castillo-Davis CI, Oshiro G, Liang D, Richards DR, Zhou Y, et al. Genetic diversity in yeast assessed with whole-genome oligonucleotide arrays. Genetics. 2003;163(1):79–89. 10.1093/genetics/163.1.79 PMC1462430 12586698

[55] Cambareri EB, Aisner R, Carbon J. Structure of the chromosome VII centromere region in Neurospora crassa: degenerate transposons and simple repeats. Mol Cell Biol. 1998;18(9):5465–77. 10.1128/mcb.18.9.5465 PMC109131 9710630

[56] Freitag M, Williams RL, Kothe GO, Selker EU. A cytosine methyltransferase homologue is essential for repeat-induced point mutation in Neurospora crassa. Proc Natl Acad Sci U S A. 2002;99(13):8802–7. 10.1073/pnas.132212899 PMC124379 12072568

[57] Heidelberg JF, Eisen JA, Nelson WC, Clayton RA, Gwinn ML, Dodson RJ, et al. DNA sequence of both chromosomes of the cholera pathogen Vibrio cholerae. Nature. 2000;406(6795):477–83. 10.1038/35020000 PMC8288016 10952301

[58] Klenk HP, Clayton RA, Tomb JF, White O, Nelson KE, Ketchum KA, et al. The complete genome sequence of the hyperthermophilic, sulphate-reducing archaeon Archaeoglobus fulgidus. Nature. 1997;390(6658):364–70. 10.1038/37052 9389475

[59] Luo R, Liu B, Xie Y, Li Z, Huang W, Yuan J, et al. SOAPdenovo2: an empirically improved memory-efficient short-read de novo assembler. GigaScience. 2012;1(1):18. 10.1186/2047-217X-1-18 PMC3626529 23587118

[60] Alkan C, Sajjadian S, Eichler EE. Limitations of next-generation genome sequence assembly. Nat Methods. 2011;8(1):61–5. 10.1038/nmeth.1527 PMC3115693 21102452

[61] Smith KM, Galazka JM, Phatale PA, Connolly LR, Freitag M. Centromeres of filamentous fungi. Chromosome Res. 2012;20(5):635–56. 10.1007/s10577-012-9290-3 PMC3409310 22752455

[62] Cleveland DW, Mao Y, Sullivan KF. Centromeres and kinetochores: from epigenetics to mitotic checkpoint signaling. Cell. 2003;112(4):407–21. 10.1016/s0092-8674(03)00115-6 12600307

[63] Meraldi P, McAinsh AD, Rheinbay E, Sorger PK. Phylogenetic and structural analysis of centromeric DNA and kinetochore proteins. Genome Biol. 2006;7(3):R23. 10.1186/gb-2006-7-3-r23 PMC1557759 16563186

[64] Sanyal K, Baum M, Carbon J. Centromeric DNA sequences in the pathogenic yeast Candida albicans are all different and unique. Proc Natl Acad Sci U S A. 2004;101(31):11374–9. 10.1073/pnas.0404318101 PMC509209 15272074

[65] Brown NA, Antoniw J, Hammond-Kosack KE. The predicted secretome of the plant pathogenic fungus Fusarium graminearum: a refined comparative analysis. Plos One. 2012. 7(4):e33731 10.1371/journal.pone.0033731 PMC3320895 22493673

[66] Galagan JE, Calvo SE, Cuomo C, Ma LJ, Wortman JR, Batzoglou S, et al. Sequencing of Aspergillus nidulans and comparative analysis with A. fumigatus and A. oryzae. Nature. 2005;438(7071):1105–15. 10.1038/nature04341 16372000

[67] Perrin RM, Fedorova ND, Bok JW, Cramer RA, Wortman JR, Kim HS, et al. Transcriptional regulation of chemical diversity in Aspergillus fumigatus by LaeA. PLoS Pathog. 2007;3(4):e50. 10.1371/journal.ppat.0030050 PMC1851976 17432932

[68] Sieber CM, Lee W, Wong P, Munsterkotter M, Mewes HW, Schmeitzl C, et al. The Fusarium graminearum genome reveals more secondary metabolite gene clusters and hints of horizontal gene transfer. PLoS One. 2014;9(10):e110311. 10.1371/journal.pone.0110311 PMC4198257 25333987

[69] Clutterbuck AJ. Genomic evidence of repeat-induced point mutation (RIP) in filamentous ascomycetes. Fungal Genet Biol. 2011;48(3):306–26. 10.1016/j.fgb.2010.09.002 20854921

[70] MIAME/Plant Compliant Gene Expression Resources for Plants and Plant Pathogens. 2014. http://www.plexdb.org/. Accessed 01 Aug 2014.

[71] King R. 2015. https://rrescloud.rothamsted.ac.uk/public.php?service=files&t=cd70482248d1f0230d3cf6346662f663. Accessed 01 Jan 2015.

[72] Urban M, Mott E, Farley T, Hammond-Kosack K. The Fusarium graminearum MAP1 gene is essential for pathogenicity and development of perithecia. Mol Plant Pathol. 2003;4(5):347–59. 10.1046/j.1364-3703.2003.00183.x 20569395

[73] Doyle J. A rapid DNA isolation procedure for small quantities of fresh leaf tissue. Phytochem Bull. 1987;19:11–5.

[74] Bentley DR, Balasubramanian S, Swerdlow HP, Smith GP, Milton J, Brown CG, et al. Accurate whole human genome sequencing using reversible terminator chemistry. Nature. 2008;456(7218):53–9. 10.1038/nature07517 PMC2581791 18987734

[75] Güldener U. MIPS version FG3.3. 2011. ftp://ftpmips.gsf.de/fungi/Fusarium/F_graminearum_PH1_v32/p3_p13839_Fus_grami_v32.scaf. Accessed 15 Jul 2014.

[76] Milne I, Stephen G, Bayer M, Cock PJ, Pritchard L, Cardle L, et al. Using Tablet for visual exploration of second-generation sequencing data. Brief Bioinform. 2013;14(2):193–202. 10.1093/bib/bbs012 22445902

[77] Tempel S. Using and understanding RepeatMasker. Methods Mol Biol. 2012;859:29–51. 10.1007/978-1-61779-603-6_2 22367864

[78] Solovyev V, Kosarev P, Seledsov I, Vorobyev D. Automatic annotation of eukaryotic genes, pseudogenes and promoters. Genome Biol. 2006;7 Suppl 1:S10 1–12. 10.1186/gb-2006-7-s1-s10 PMC1810547 16925832

[79] Stanke M, Schoffmann O, Morgenstern B, Waack S. Gene prediction in eukaryotes with a generalized hidden Markov model that uses hints from external sources. BMC Bioinformatics. 2006;7:62. 10.1186/1471-2105-7-62 PMC1409804 16469098

[80] Lukashin AV, Borodovsky M. GeneMark.hmm: new solutions for gene finding. Nucleic Acids Res. 1998;26(4):1107–15. 10.1093/nar/26.4.1107 PMC147337 9461475

[81] Korf I. Gene finding in novel genomes. BMC Bioinformatics. 2004;5:59. 10.1186/1471-2105-5-59 PMC421630 15144565

[82] Lowe TM, Eddy SR. tRNAscan-SE: a program for improved detection of transfer RNA genes in genomic sequence. Nucleic Acids Res. 1997;25(5):955–64. 10.1093/nar/25.5.955 PMC146525 9023104

[83] Nawrocki EP, Eddy SR. Infernal 1.1: 100-fold faster RNA homology searches. Bioinformatics. 2013;29(22):2933–5. 10.1093/bioinformatics/btt509 PMC3810854 24008419

[84] Softberry. 2015. www.SoftBerry.com. Accessed 24 Aug 2014.

[85] Horton P, Park KJ, Obayashi T, Fujita N, Harada H, Adams-Collier CJ, et al. WoLF PSORT: protein localization predictor. Nucleic Acids Res. 2007;35(Web Server issue):W585–7. 10.1093/nar/gkm259 PMC1933216 17517783

[86] Eisenhaber B, Wildpaner M, Schultz CJ, Borner GH, Dupree P, Eisenhaber F. Glycosylphosphatidylinositol lipid anchoring of plant proteins. Sensitive prediction from sequence- and genome-wide studies for Arabidopsis and rice. Plant Physiol. 2003;133(4):1691–701. 10.1104/pp.103.023580 PMC300724 14681532

[87] Li H, Durbin R. Fast and accurate short read alignment with Burrows-Wheeler transform. Bioinformatics. 2009;25(14):1754–60. 10.1093/bioinformatics/btp324 PMC2705234 19451168

[88] Li H, Handsaker B, Wysoker A, Fennell T, Ruan J, Homer N, et al. The Sequence Alignment/Map format and SAMtools. Bioinformatics. 2009;25(16):2078–9. 10.1093/bioinformatics/btp352 PMC2723002 19505943

