# Supplementary material for: The completed genome sequence of the pathogenic ascomycete fungus Fusarium graminearum
Source: BMC Genomics. 2015 Jul 22;16(1):544. doi: 10.1186/s12864-015-1756-1 (PMC4511438; doi:10.1186/s12864-015-1756-1)

Additional file 1. Comparisons of H3K9me3 and H3K27me3 binding to the MIPS reference with an alignment superimposed of MIPS vs RRes. The only chromosome centromere to show H3K9me3 binding (normally associated with chromatin) in the MIPS reference from the *Connolly et al* study is chromosome 3 but this is explained due to chromosome 3 being the only sequence in the MIPS version that had part of the centromere sequence complete. Further studies are likely to show this.

Chromosome3, alignment shows the MIPS version had part of the centromere sequence hence why see some H3K9me3 binding.

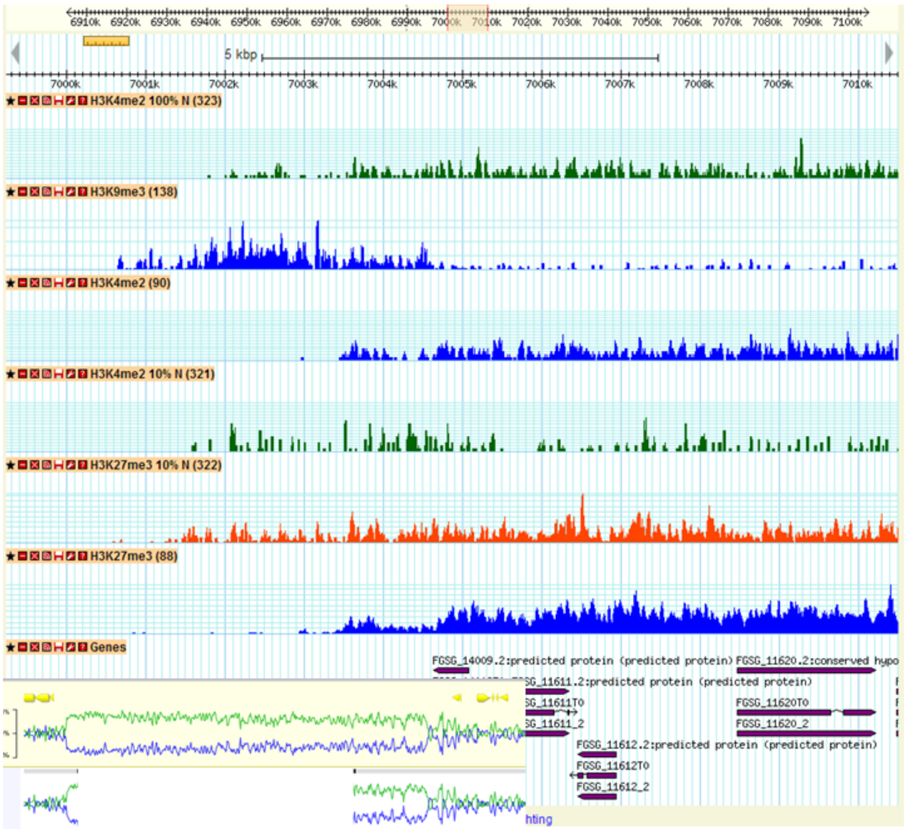

Chromosome4, alignment shows the MIPS version did not have part of the centromere sequence hence why no H3K9me3 binding is observed.

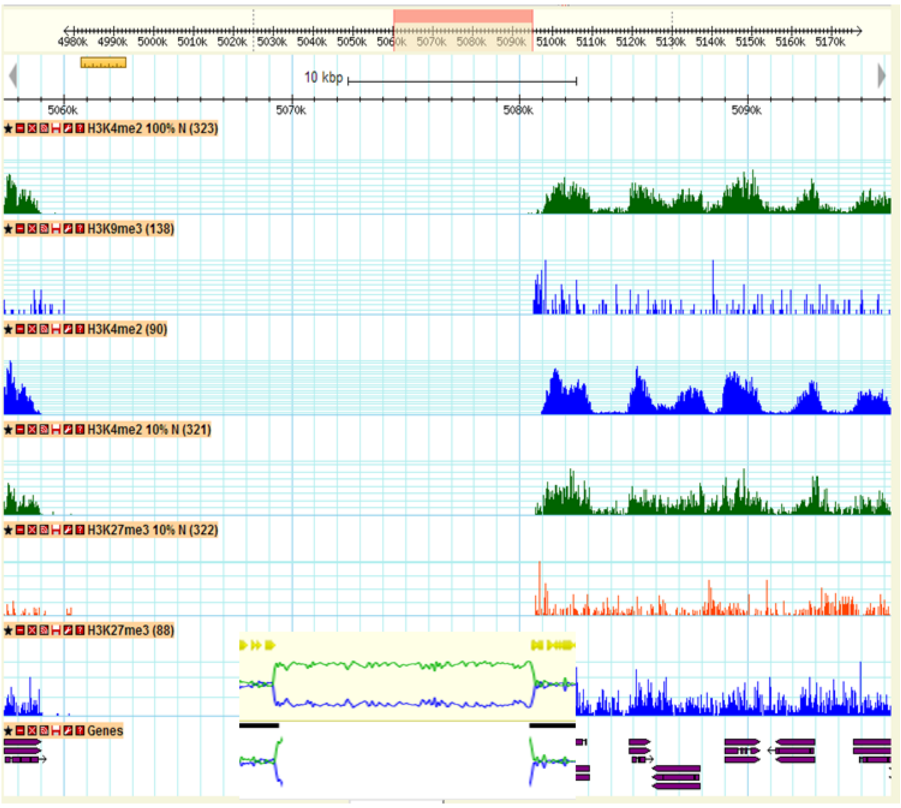

Chromosome2, alignment shows the MIPS version did not have part of the centromere sequence hence why no H3K9me3 binding is observed.

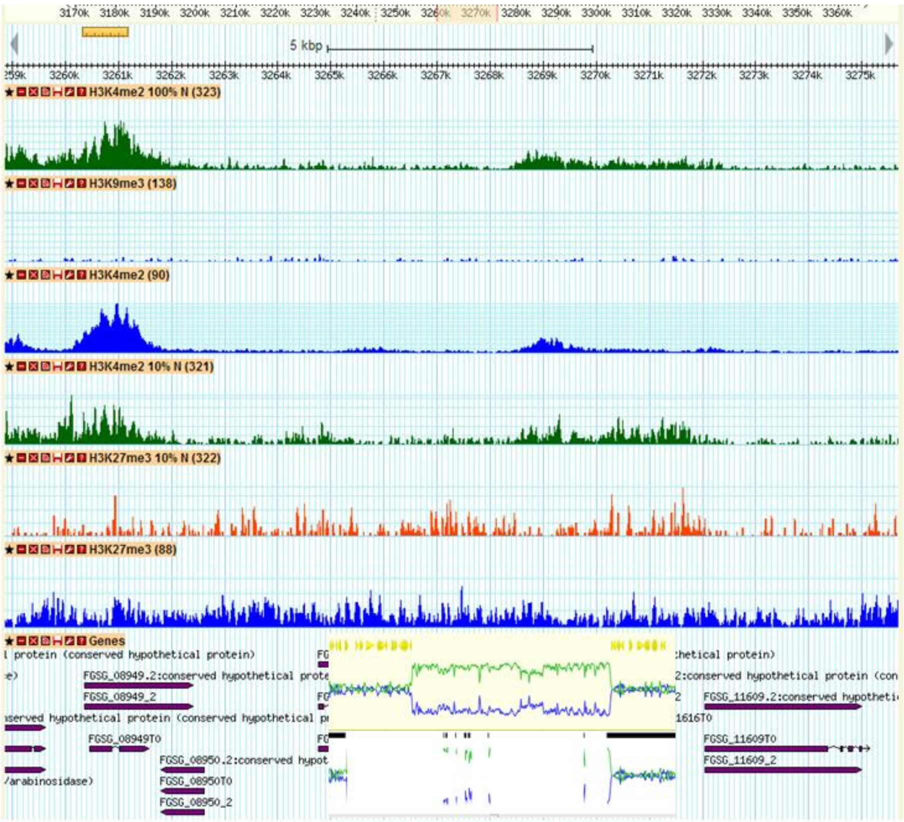

Chromosome1, alignment shows the MIPS version did not have part of the centromere sequence hence why no H3K9me3 binding is observed.

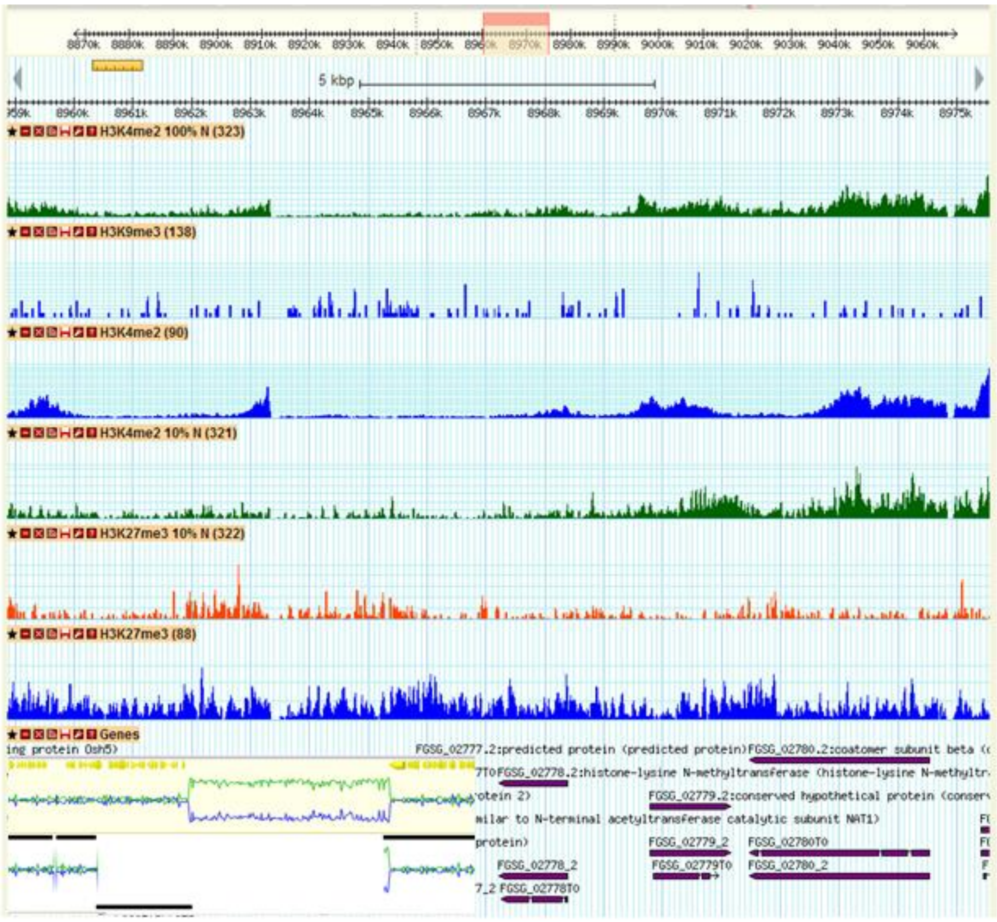

Supplement: Additional file 1: — Figures showing H3K9me3 binding in the MIPS reference with alignments of RRes and MIPs reference to highlight where centromeric sequence was present in the Connolly et al. study. [file 12864_2015_1756_MOESM1_ESM.pdf]
