# Supplementary material for: The completed genome sequence of the pathogenic ascomycete fungus Fusarium graminearum
Source: BMC Genomics. 2015 Jul 22;16(1):544. doi: 10.1186/s12864-015-1756-1 (PMC4511438; doi:10.1186/s12864-015-1756-1)
Supplement: Additional file 2: — A table listing the gene models in RRes v4.0 deleted from the MIPS annotation. [file 12864_2015_1756_MOESM2_ESM.pdf]

Additional file 2. Deleted gene models (bp length in brackets, *isoform).

Chr 1	Chr 2	Chr 3	Chr 4
FGSG_11749 (32)	FGSG_15223 (58)	FGSG_12844 (113)	FGSG_15195 (57)
FGSG_15045 (66)	FGSG_15495 (78)		FGSG_16834 (21)
FGSG_15100 (55)	FGSG_13453 (151)		FGSG_15417 (62)
FGSG_15125 (59)	FGSG_13448 (133)		
FGSG_16203 (79)	FGSG_15191 (51)		
FGSG_01500 (105)			
FGSG_17612 (1039)*			
FGSG_00674 (167)*			
FGSG_15616 (86)			
