# Supplementary material for: The completed genome sequence of the pathogenic ascomycete fungus Fusarium graminearum
Source: BMC Genomics. 2015 Jul 22;16(1):544. doi: 10.1186/s12864-015-1756-1 (PMC4511438; doi:10.1186/s12864-015-1756-1)
Supplement: Additional file 3: — A table of RRes v4.0 and MIPS v3.2 binned protein lengths. [file 12864_2015_1756_MOESM3_ESM.pdf]

Additional file 3. Length of proteins of RRes and MIPS v3.2 sets

	MIPS		RRes	
Length	No. of Proteins	% Proteins	No. of Proteins	% Proteins
Proteins < 50 aa	35	0.30%	68	0.48%
Proteins 50-149 aa	2143	15.50%	2353	16.61%
Proteins 150-299 aa	2980	21.60%	2920	20.62%
Proteins 300-999 aa	7783	56.30%	7899	55.77%
Proteins 1000-3000 aa	861	6.20%	899	6.35%
Proteins > 3000 aa	24	0.20%	25	0.18%
