# Supplementary material for: The completed genome sequence of the pathogenic ascomycete fungus Fusarium graminearum
Source: BMC Genomics. 2015 Jul 22;16(1):544. doi: 10.1186/s12864-015-1756-1 (PMC4511438; doi:10.1186/s12864-015-1756-1)
Supplement: Additional file 4: — A table of RRes v4.0 and MIPS exons per transcript. [file 12864_2015_1756_MOESM4_ESM.pdf]

Additional file 4. Exons per transcript distribution using Eval.

Total number of exons /gene model	RRes	MIPS v3.2	RRes diff	RRes % of total gene models	MIPS % of total gene models
1	3536	3250	+286	24.96	23.51
2	4206	4130	+76	29.70	29.87
3	2961	2957	+4	20.91	21.39
4	1616	1646	-30	11.41	11.91
5	857	866	-9	6.05	6.26
6	440	448	-8	3.11	3.24
7	203	210	-7	1.43	1.52
8	144	127	+17	1.02	0.92
9	90	85	+5	0.64	0.61
10	44	46	-2	0.31	0.33
11	30	27	+3	0.21	0.20
12	17	15	+2	0.12	0.11
13	6	3	+3	0.04	0.02
14	5	10	-5	0.04	0.07
15	4	3	+1	0.03	0.02
16	1	1	0	0.01	0.01
17	2	0	+2	0.01	0.00
18	0	0	0	0.00	0.00
19	1	1	0	0.01	0.01
20	0	0	0	0.00	0.00
21	1	1	0	0.01	0.01
