# Supplementary material for: The completed genome sequence of the pathogenic ascomycete fungus Fusarium graminearum
Source: BMC Genomics. 2015 Jul 22;16(1):544. doi: 10.1186/s12864-015-1756-1 (PMC4511438; doi:10.1186/s12864-015-1756-1)
Supplement: Additional file 5: — A table listing the BLAST and GO annotations of the RRes v4.0 new gene prediction subset. [file 12864_2015_1756_MOESM5_ESM.pdf]

Additional file 5. New gene predictions blast and GO ID's annotations using Blast2Go. DOCX format.

SeqName FGRRES_	Hit-Description	GO-Group	GO-ID	Term
20004	hypothetical protein FG00057.1	F	GO:0016491	oxidoreductase activity
20006	related to micromolar calcium activated neutral protease 1	C	GO:0005622	intracellular
20006	related to micromolar calcium activated neutral protease 1	P	GO:0006508	proteolysis
20006	related to micromolar calcium activated neutral protease 1	F	GO:0004198	calcium-dependent cysteine-type endopeptidase activity
20036	hypothetical protein FOVG_01478	F	GO:0004872	receptor activity
20036	hypothetical protein FOVG_01478	P	GO:0007275	multicellular organismal development
20036	hypothetical protein FOVG_01478	C	GO:0016020	membrane
20048	hypothetical protein FG05_30608	F	GO:0000981	sequence-specific DNA binding RNA polymerase II transcription factor activity
20048	hypothetical protein FG05_30608	P	GO:0006357	regulation of transcription from RNA polymerase II promoter
20048	hypothetical protein FG05_30608	C	GO:0005634	nucleus
20048	hypothetical protein FG05_30608	F	GO:0008270	zinc ion binding
20056	epoxide hydrolase	F	GO:0016787	hydrolase activity
20056	epoxide hydrolase	P	GO:0055114	oxidation-reduction process
20056	epoxide hydrolase	F	GO:0016706	oxidoreductase activity, acting on paired donors, with incorporation or reduction of molecular oxygen, 2-oxoglutarate as one donor, and incorporation of one atom each of oxygen into both donors
20063	v-type h+-transporting atpase subunit h	F	GO:0015078	hydrogen ion transmembrane transporter activity
20063	v-type h+-transporting atpase subunit h	F	GO:0042625	ATPase activity, coupled to transmembrane movement of ions
20063	v-type h+-transporting atpase subunit h	C	GO:0000329	fungal-type vacuole membrane

20063	v-type h+-transporting atpase subunit h	P	GO:0000032	cell wall mannoprotein biosynthetic process
20063	v-type h+-transporting atpase subunit h	P	GO:0006461	protein complex assembly
20063	v-type h+-transporting atpase subunit h	P	GO:0007035	vacuolar acidification
20063	v-type h+-transporting atpase subunit h	C	GO:0000220	vacuolar proton-transporting V-type ATPase, V0 domain
20063	v-type h+-transporting atpase subunit h	P	GO:0015991	ATP hydrolysis coupled proton transport
20064	hypothetical protein FG01998.1	F	GO:0008270	zinc ion binding
20064	hypothetical protein FG01998.1	F	GO:0016832	aldehyde-lyase activity
20064	hypothetical protein FG01998.1	P	GO:0005975	carbohydrate metabolic process
20067	related to permease of the major facilitator superfamily	C	GO:0016021	integral component of membrane
20067	related to permease of the major facilitator superfamily	F	GO:0003723	RNA binding
20067	related to permease of the major facilitator superfamily	F	GO:0008270	zinc ion binding
20067	related to permease of the major facilitator superfamily	P	GO:0055085	transmembrane transport
20078	hypothetical protein FG05_30155	C	GO:0016021	integral component of membrane
20101	hypothetical protein FG10427.1	F	GO:0050660	2ydrol adenine dinucleotide binding
20101	hypothetical protein FG10427.1	F	GO:0008762	UDP-N-acetylmuramate dehydrogenase activity
20101	hypothetical protein FG10427.1	P	GO:0055114	oxidation-reduction process
20105	arylsulfatase precursor	F	GO:0016787	hydrolase activity
20105	arylsulfatase precursor	P	GO:0008152	metabolic process
20107	hypothetical protein FOTG_17375	F	GO:0003824	catalytic activity

20107	hypothetical protein FOTG_17375	P	GO:0009116	nucleoside metabolic process
20116	glucose transporter rco-3	C	GO:0016021	integral component of membrane
20116	glucose transporter rco-3	F	GO:0022891	substrate-specific transmembrane transporter activity
20116	glucose transporter rco-3	P	GO:0055085	transmembrane transport
20116	glucose transporter rco-3	P	GO:0008643	carbohydrate transport
20126	hypothetical protein FG05_30313	C	GO:0005956	protein kinase CK2 complex
20126	hypothetical protein FG05_30313	P	GO:0045859	regulation of protein kinase activity
20126	hypothetical protein FG05_30313	P	GO:0006357	regulation of transcription from RNA polymerase II promoter
20126	hypothetical protein FG05_30313	F	GO:0008270	zinc ion binding
20126	hypothetical protein FG05_30313	F	GO:0000981	sequence-specific DNA binding RNA polymerase II transcription factor activity
20126	hypothetical protein FG05_30313	F	GO:0003677	DNA binding
20126	hypothetical protein FG05_30313	C	GO:0005634	nucleus
20126	hypothetical protein FG05_30313	F	GO:0019887	protein kinase regulator activity
20151	hypothetical protein FG08862.1	F	GO:0008270	zinc ion binding
20151	hypothetical protein FG08862.1	F	GO:0003676	nucleic acid binding
20168	hypothetical protein FG05_03154	P	GO:0055114	oxidation-reduction process
20168	hypothetical protein FG05_03154	P	GO:0006357	regulation of transcription from RNA polymerase II promoter
20168	hypothetical protein FG05_03154	F	GO:0008270	zinc ion binding
20168	hypothetical protein FG05_03154	F	GO:0000981	sequence-specific DNA binding RNA polymerase II transcription factor activity
20168	hypothetical protein FG05_03154	F	GO:0016491	oxidoreductase activity
20168	hypothetical protein FG05_03154	C	GO:0005634	nucleus
20171	aldolase	P	GO:0006725	cellular aromatic compound metabolic process
20171	aldolase	F	GO:0016830	carbon-carbon lyase activity
20172	cytochrome p450 oxidoreductase	F	GO:0016708	oxidoreductase activity, acting on paired donors, with incorporation or reduction of molecular oxygen,

				NAD(P)H as one donor, and incorporation of two atoms of oxygen into one donor
20172	cytochrome p450 oxidoreductase	P	GO:0019439	aromatic compound catabolic process
20172	cytochrome p450 oxidoreductase	F	GO:0004497	monooxygenase activity
20172	cytochrome p450 oxidoreductase	F	GO:0005506	iron ion binding
20172	cytochrome p450 oxidoreductase	F	GO:0051537	2 iron, 2 sulfur cluster binding
20172	cytochrome p450 oxidoreductase	P	GO:0055114	oxidation-reduction process
20176	pathogenicity protein	F	GO:0005215	transporter activity
20182	hypothetical protein FG05_35086	P	GO:0006357	regulation of transcription from RNA polymerase II promoter
20182	hypothetical protein FG05_35086	F	GO:0008270	zinc ion binding
20182	hypothetical protein FG05_35086	F	GO:0000981	sequence-specific DNA binding RNA polymerase II transcription factor activity
20182	hypothetical protein FG05_35086	F	GO:0003677	DNA binding
20182	hypothetical protein FG05_35086	C	GO:0005634	nucleus
20189	homocitrate synthase	F	GO:0004410	homocitrate synthase activity
20189	homocitrate synthase	C	GO:0016021	integral component of membrane
20189	homocitrate synthase	C	GO:0005634	nucleus
20189	homocitrate synthase	C	GO:0005739	mitochondrion
20189	homocitrate synthase	P	GO:0019878	lysine biosynthetic process via aminoadipic acid
20236	cytochrome p450	P	GO:0006357	regulation of transcription from RNA polymerase II promoter
20236	cytochrome p450	F	GO:0008270	zinc ion binding
20236	cytochrome p450	F	GO:0000981	sequence-specific DNA binding RNA polymerase II transcription factor activity
20236	cytochrome p450	F	GO:0003677	DNA binding
20236	cytochrome p450	C	GO:0005634	nucleus
20239	rta1 domain-containing protein	P	GO:0006357	regulation of transcription from RNA polymerase II promoter
20239	rta1 domain-containing protein	F	GO:0008270	zinc ion binding
20239	rta1 domain-containing protein	F	GO:0004553	hydrolase activity, 4ydrolysing O-glycosyl compounds

20239	rta1 domain-containing protein	F	GO:0000981	sequence-specific DNA binding RNA polymerase II transcription factor activity
20239	rta1 domain-containing protein	F	GO:0016758	transferase activity, transferring hexosyl groups
20239	rta1 domain-containing protein	C	GO:0005634	nucleus
20239	rta1 domain-containing protein	P	GO:0030259	lipid glycosylation
20242	hypothetical protein FG05_04947	F	GO:0046872	metal ion binding
20242	hypothetical protein FG05_04947	F	GO:0004672	protein kinase activity
20242	hypothetical protein FG05_04947	P	GO:0006468	protein phosphorylation
20242	hypothetical protein FG05_04947	F	GO:0005524	ATP binding
20245	hypothetical protein FG05020.1	F	GO:0008270	zinc ion binding
20247	zinc finger protein ozf-like protein	F	GO:0046872	metal ion binding
20251	---NA---	F	GO:0016491	oxidoreductase activity
20251	---NA---	P	GO:0055114	oxidation-reduction process
20255	rpp14 family	F	GO:0004540	ribonuclease activity
20255	rpp14 family	P	GO:0090501	RNA phosphodiester bond hydrolysis
20255	rpp14 family	P	GO:0008033	tRNA processing
20264	phosphatidylinositol 4-kinase	P	GO:0046854	phosphatidylinositol phosphorylation
20264	phosphatidylinositol 4-kinase	F	GO:0004430	1-phosphatidylinositol 4-kinase activity
20264	phosphatidylinositol 4-kinase	P	GO:0006661	phosphatidylinositol biosynthetic process
20264	phosphatidylinositol 4-kinase	P	GO:0048015	phosphatidylinositol-mediated signaling
20264	phosphatidylinositol 4-kinase	F	GO:0005488	binding
20283	hypothetical protein FPSE_02973	P	GO:0009306	protein secretion
20289	fungal transcriptional regulatory protein	F	GO:0000981	sequence-specific DNA binding RNA polymerase II transcription factor activity
20289	fungal transcriptional regulatory protein	P	GO:0006357	regulation of transcription from RNA polymerase II promoter
20289	fungal transcriptional regulatory protein	C	GO:0005634	nucleus
20289	fungal transcriptional regulatory protein	F	GO:0008270	zinc ion binding

20292	hypothetical protein FG06162.1	F	GO:0004190	aspartic-type endopeptidase activity
20292	hypothetical protein FG06162.1	P	GO:0006508	proteolysis
20294	serine threonine protein kinase	F	GO:0004672	protein kinase activity
20294	serine threonine protein kinase	P	GO:0006468	protein phosphorylation
20294	serine threonine protein kinase	F	GO:0005524	ATP binding
20304	meiosis protein mei2	F	GO:0003676	nucleic acid binding
20304	meiosis protein mei2	F	GO:0000166	nucleotide binding
20323	hypothetical protein FG11465.1	F	GO:0020037	heme binding
20323	hypothetical protein FG11465.1	F	GO:0016705	oxidoreductase activity, acting on paired donors, with incorporation or reduction of molecular oxygen
20323	hypothetical protein FG11465.1	F	GO:0004497	monooxygenase activity
20323	hypothetical protein FG11465.1	F	GO:0005506	iron ion binding
20323	hypothetical protein FG11465.1	P	GO:0055114	oxidation-reduction process
20326	hypothetical protein FG05_35376	F	GO:0043565	sequence-specific DNA binding
20326	hypothetical protein FG05_35376	F	GO:0003700	sequence-specific DNA binding transcription factor activity
20326	hypothetical protein FG05_35376	P	GO:0006355	regulation of transcription, DNA-templated
20327	alpha-n-arabinofuranosidase	F	GO:0046556	alpha-L-arabinofuranosidase activity
20327	alpha-n-arabinofuranosidase	C	GO:0005576	extracellular region
20327	alpha-n-arabinofuranosidase	P	GO:0046373	L-arabinose metabolic process
20327	alpha-n-arabinofuranosidase	F	GO:0030248	cellulose binding
20330	related to galactoside o-acetyltransferase	F	GO:0016407	acetyltransferase activity
20330	related to galactoside o-acetyltransferase	P	GO:0008152	metabolic process
20373	alkylated dna repair protein alkb like protein 8	P	GO:0055114	oxidation-reduction process
20373	alkylated dna repair protein alkb like protein 8	F	GO:0016706	oxidoreductase activity, acting on paired donors, with incorporation or reduction of molecular oxygen, 2-oxoglutarate as one donor, and

				incorporation of one atom each of oxygen into both donors
20377	cutinase	C	GO:0005576	extracellular region
20377	cutinase	P	GO:0008152	metabolic process
20377	cutinase	F	GO:0050525	cutinase activity
20397	hypothetical protein FPSE_09629	P	GO:0032259	methylation
20397	hypothetical protein FPSE_09629	F	GO:0008168	methyltransferase activity
20406	serine threonine protein kinase	P	GO:0006468	protein phosphorylation
20406	serine threonine protein kinase	F	GO:0005524	ATP binding
20406	serine threonine protein kinase	P	GO:0046677	response to antibiotic
20406	serine threonine protein kinase	F	GO:0004674	protein serine/threonine kinase activity
20409	gag protein	F	GO:0003676	nucleic acid binding
20409	gag protein	F	GO:0008270	zinc ion binding
20411	histone h4	C	GO:0000786	nucleosome
20411	histone h4	P	GO:0043581	mycelium development
20411	histone h4	F	GO:0003677	DNA binding
20411	histone h4	P	GO:0006334	nucleosome assembly
20411	histone h4	F	GO:0046982	protein heterodimerization activity
20411	histone h4	P	GO:0006352	DNA-templated transcription, initiation
20411	histone h4	C	GO:0005634	nucleus
