# Supplementary material for: The completed genome sequence of the pathogenic ascomycete fungus Fusarium graminearum
Source: BMC Genomics. 2015 Jul 22;16(1):544. doi: 10.1186/s12864-015-1756-1 (PMC4511438; doi:10.1186/s12864-015-1756-1)
Supplement: Additional file 6: — A description and table listing the RRes secretome gene ID’s and those unique to the earlier Brown et al. [ 42 ] vs. RRes v4.0 secretome. [file 12864_2015_1756_MOESM6_ESM.pdf]

Additional file 6. Description of secretome methodology.

RRes secretome prediction found signal domain (RRes:2189), removal of sequences with transmembrane domains (RRes:1776) using Interproscan, exclusion of proteins smaller than 20, and the use of ProtComp (Version 9.0) LocDB and PotLocDB to exclude GPI anchored membrane proteins and other non-extracellular loci proteins (RRes:1602), followed by WoLfPSort [28] (extracellular score > 17, WoLfPSort) (RRes:705)) to identify final destination and big-PI [29] to further remove GPI-anchored proteins (RRes:616).

For the 412 new genes the use of blast NCBI 04/09/14 and Interproscan in blast2go revealed, after the blast2go filtering algorithm, 147 of the new gene protein sequences had blast hits using an e-value of 0.001 before blast2go filtering, and 115 had Interproscan functional annotation. After merging of GO terms from Interproscan, 99 Level 2 biological process and 64 molecular function GO terms were identified, see Table-4. Of the 412, 44 of the new genes have signal domains but 6 have transmembrane of those 38, 12 passed the extracellular filtering using ProtComp (Version 9.0) and WoLfPSort (extracellular score > 17) could be new secretome proteins. The top-hit species blast results are shown in supplementary Table-12 which shows that many of the new genes have similar gene models reported in other genome assemblies/annotations of *F. graminearum* or in lesser cases other fungi.

Complete list of FGRRES_gene ID's of predicted secretome and those uniquely predicted vs the *Brown et al* secretome set. *(MIPS V30 ID) gene identified in *Brown et al* secretome which used MIPS V30 gene models, **(RRes ID) gene model from MIPS version V32. 144 of the RRes predictions were confirmed using proteomics from *Yang et al* and *Paper et el*. [£]30 of the 70 proteins identified in the *Yang et al* study from proteins identified in *F. graminearum* culture supernatants after growth in wheat or barley flour-containing medium were confirmed in the in silica predictions. [$]137 of the 286

from the extracellular proteins in vitro and in planta from the pathogenic fungus *F. graminearum* in the *Paper et al* study were confirmed in the in silica predictions.

RRes (all) (n=616)	Identified in both studies (n= 515)	RRes (unique) (n=101)	Brown *et al.*, (2012) (unique) (n=59)
00002	00002	00072	00129
00006	00006	00571	00742
00009	00009	01351	00806
00023	00023	01711	01437
00028$	00028	01846	01588
00029	00029	01993	02348
00060$	00060	02190	02685
00061	00061	02204	02976
00062	00062	02293	03406
00072	00100	02328	03495
00100$	00111	02351	03574
00111	00112	02385	03816
00112	00114	02408	03827
00114$	00131	02721	03842
00131	00184	03114	03975
00184$	00230	03157	04372
00230	00260	03662	04397
00260	00294	03713	04521
00294	00411	04345	04597
00411	00569	04758	04699
00569	00642	04781	04824
00571$	00783	05345	04900
00642	00847	05628	05757
00783	00987	05835	06479
00847	00989	06549	06572
00987	01239	06888	06605
00989$	01240	07010	06616
01239	01283	07772	06712
01240	01570	08003	06741(16708)**
01283	01595	08493	07556(16845)**
01351	01598	09522	07678
01570	01603	10604	07871
01595$	01607	11077	07944
01598	01621	11231	08136
01603	01636	11333	08415
01607$	01660	11350	08454(17031)**

01621[$]	01674	11361	08549
01636	01685	12486	08583(17009)**
01660	01688	12652	09291
01674	01728	13462	09307
01685	01748	15885	09390
01688	01754	15937	09742
01711	01763	15958	10525
01728	01776	16044	10595
01748[$]	01778	16066	11164
01754	01803	16175	11236
01763	01815	16196	11472
01776	01818	16234	11517(16047)**
01778	01829	16333	11645
01803	01831	16334	11745(15729)**
01815	01872	16408	12337
01829[$]	01982	16459	12414(16243)**
01831	01988	16547	12439
01846	02015	16584	13583(17212)**
01872	02059	16623	13883
01982	02154	16668	13924(17524)**
01988[$]	02202	16697	13992(17575)**
01993[$]	02228	16759	15260(16423)**
02015	02255	16883	15448
02059[$]	02263	16898	
02190	02269	17022	
02202	02309	17115	
02204[$]	02314	17419	
02228	02330	17425	
02255[$]	02332	17434	
02263	02339	17455	
02269	02354	17468	
02293	02360	17469	
02309[$]	02378	17494	
02314	02422	17545	
02328	02448	17566	
02330	02651	20027	
02332	02658	20104	
02339[$]	02674	20176	
02351	02686	20208	
02354[$]	02841	20240	
02360[$]	02888	20306	
02378	02890	20325	

02385	02893	20327	
02408	02899	20377	
02422	02917	20390	
02448	02918	20400	
02651	02933	20407	
02658[£$]	02962	02909_M	
02674	02977	04165_M	
02686	02987	04374_M	
02721[$]	02999	04756_M	
02841	03002	07126_M	
02888	03003	07686_M	
02890	03034	08074_M	
02893	03035	09143_M	
02899	03049	10563_M	
02917	03050	10607_M	
02918[$]	03052	10776_M	
02933	03054	11513_M	
02962	03096	12405_M	
02987	03109	13975_M	
02999	03121	13987_M	
03002[$]	03122	15136_M	
03003[£$]	03123	15714_M	
03035[£$]	03130	17388_M	
03049[$]	03131		
03050	03143		
03052[$]	03156		
03054[$]	03194		
03096	03209		
03109	03212		
03114[$]	03217		
03121	03243		
03122	03274		
03123[$]	03275		
03130	03304		
03131[$]	03307		
03143[$]	03315		
03156	03331		
03157	03334		
03194[$]	03343		
03209	03348		
03212[$]	03365		
03217	03371		

03243[$]	03379		
03274	03384		
03275	03387		
03304	03394		
03307	03402		
03315[$]	03432		
03334[$]	03436		
03343[$]	03445		
03348	03457		
03365[£$]	03463		
03371	03467		
03379[$]	03483		
03384	03507		
03394	03521		
03402	03526		
03432	03529		
03436	03531		
03445	03532		
03457	03544		
03463	03569		
03467[$]	03570		
03483[$]	03583		
03507	03584		
03521	03585		
03526	03598		
03529	03599		
03531	03600		
03532	03601		
03569	03609		
03583	03612		
03584[$]	03614		
03585	03616		
03598[$]	03624		
03599	03628		
03600[$]	03629		
03601	03632		
03609[$]	03640		
03612	03674		
03614	03687		
03616	03695		
03624[$]	03708		
03628[$]	03724		

03629	03748		
03632[$]	03769		
03640	03784		
03662[$]	03795		
03695[$]	03813		
03708	03820		
03713	03846		
03724	03858		
03748	03865		
03769	03867		
03784	03883		
03795[$]	03893		
03813[$]	03894		
03820	03896		
03846	03901		
03858	03904		
03865[$]	03905		
03883[£]	03908		
03893	03911		
03896	03916		
03901	03922		
03904	03944		
03905[$]	03954		
03908[$]	03958		
03911	03960		
03916	03969		
03922	03972		
03944	03986		
03954[$]	04029		
03958	04060		
03960	04074		
03969	04146		
03972	04239		
03986[$]	04380		
04029	04429		
04060	04434		
04074[£$]	04439		
04146	04471		
04345	04503		
04380	04504		
04429	04527		
04434	04546		

04439	04563		
04471	04583		
04503	04620		
04504	04624		
04527[£$]	04646		
04546[$]	04656		
04563	04678		
04583[$]	04681		
04620	04685		
04624	04689		
04656	04704		
04678	04732		
04685	04735		
04689	04739		
04704	04740		
04732[£]	04743		
04735[$]	04744		
04739	04745		
04740	04768		
04743	04817		
04744	04818		
04745	04841		
04758[£$]	04848		
04768[$]	04858		
04781	04864		
04817	04895		
04818	04953		
04841	04980		
04848[$]	05046		
04858	05052		
04864[$]	05163		
04895	05245		
04953	05341		
04980	05609		
05046	05719		
05052	05763		
05163	05797		
05245	05841		
05341	05847		
05345	05851		
05609	05906		
05628	05933		

05719	05947		
05763	05970		
05797[£$]	05983		
05835	06017		
05841	06087		
05847	06110		
05851[$]	06117		
05906	06278		
05933	06437		
05947	06443		
05970	06451		
05983	06452		
06017[$]	06463		
06087	06465		
06110	06466		
06117	06469		
06278[£]	06497		
06443	06506		
06451	06610		
06452[$]	06612		
06463[$]	06733		
06465[$]	06775		
06466[£$]	06895		
06469	06993		
06497	07207		
06506	07221		
06549[£$]	07238		
06610	07435		
06612	07551		
06733	07569		
06775	07608		
06888	07625		
06895	07629		
06993[£$]	07639		
07010	07661		
07207	07684		
07221	07695		
07238	07698		
07435	07699		
07551[$]	07721		
07569[$]	07728		
07608[$]	07755		

07625	07775		
07629	07794		
07639[$]	07797		
07661	07829		
07695[$]	07838		
07698	07899		
07699	07921		
07721	07988		
07728	07993		
07755	07996		
07772[$]	08002		
07775[£$]	08011		
07794	08021		
07797	08026		
07829	08041		
07838[£$]	08048		
07899	08085		
07921[$]	08090		
07988[$]	08094		
07993	08115		
08002	08116		
08003	08122		
08011[$]	08150		
08026	08175		
08048[$]	08180		
08085	08196		
08094	08210		
08115	08238		
08116	08265		
08122[$]	08344		
08150	08387		
08175	08464		
08180	08824		
08196	08825		
08210	08907		
08238[$]	08958		
08265[£$]	08978		
08344	08987		
08387	09032		
08464	09066		
08493	09071		
08824	09085		

08825[$]	09093		
08907	09099		
08958	09109		
08978	09118		
08987	09127		
09066	09132		
09071	09134		
09093	09142		
09099	09181		
09109	09345		
09118	09353		
09127	09358		
09132	09382		
09134	09445		
09142	09475		
09181	09586		
09345	09646		
09353	09821		
09358	10206		
09382	10212		
09445	10316		
09475[$]	10357		
09522[$]	10435		
09586	10495		
09646	10500		
09821	10543		
10206	10551		
10212[$]	10554		
10316[$]	10561		
10357	10587		
10435	10592		
10495	10594		
10500	10609		
10543[$]	10611		
10551	10670		
10554	10675		
10561	10676		
10587	10677		
10592	10712		
10604	10713		
10609[$]	10729		
10611[$]	10972		

10670[$]	10982		
10675[$]	10986		
10676	10998		
10677[$]	10999		
10712[$]	11006		
10713	11008		
10729	11011		
10972	11032		
10982[$]	11033		
10986	11036		
10998	11037		
10999[$]	11046		
11006	11047		
11008[£]	11048		
11011[$]	11052		
11032	11066		
11033[£$]	11094		
11036[$]	11095		
11037[£$]	11097		
11046	11101		
11047	11106		
11048[$]	11112		
11052	11136		
11066	11143		
11077	11156		
11094	11163		
11095	11166		
11097[£$]	11169		
11106	11184		
11112[$]	11190		
11136	11204		
11143	11205		
11156	11206		
11163[$]	11208		
11166	11225		
11169[£$]	11227		
11184[£$]	11228		
11190[$]	11229		
11204[£]	11232		
11205[$]	11238		
11206	11249		
11208[$]	11276		

11225	11280		
11227	11296		
11228[$]	11304		
11229	11315		
11231	11318		
11232	11326		
11238	11348		
11249[$]	11360		
11276	11366		
11280[£$]	11379		
11296	11386		
11304[$]	11428		
11315	11487		
11318	11488		
11326[$]	11493		
11348[$]	11496		
11350	11516		
11360	11528		
11361	11548		
11366[$]	11559		
11379	11602		
11386[£$]	11647		
11428[$]	11648		
11487[$]	11675		
11488	11715		
11493	12067		
11496	12081		
11516	12119		
11528	12142		
11548[$]	12206		
11559	12210		
11602	12214		
11647	12251		
11675	12300		
11715	12369		
12067	12390		
12081	12434		
12119	12504		
12142	12514		
12206	12548		
12210	12551		
12214	12554		

12251	12591		
12300	12622		
12369[£]	12644		
12390	12835		
12434	12918		
12486	13189		
12504	13245		
12514	13412		
12548	13436		
12551	13443		
12554	13450		
12591	13505		
12622	13515		
12644	13628		
12652	13692		
12835	13876		
12918	13958		
13189	14020		
13412	15003		
13443	15048		
13450	15123		
13462	15142		
13505	15251		
13515	15437		
13628	15469		
13692	15661		
13876	15679(00031)*		
13958	16013(02551)*		
15003	16371(02898)*		
15048	16353(03012)*		
15123	16328(03095)*		
15142	16324(03129)*		
15251	16542(12807)*		
15437	17151(09046)*		
15469	17535(11490)*		
15661	17084(08213)*		
15679	17063(08289)*		
15885	17119(08007)*		
15937	16930(07934)*		
15958	16877(07691)*		
15982	16148(04097)*		
16013	16104(04501)*		
16044	16074(04684)*		

16066	16635(06332)*		
16074	16657(06438)*		
16104	17402(10615)*		
16148	17405(10622)*		
16163	17424(10656)*		
16175	17462(11170)*		
16196	15982(12127)*		
16234	16163(12326)*		
16324	17459(13840)*		
16328	12545_16364_M(12544)*		
16333	16312(12492)*		
16334	16111(12256)*		
16353	16018(12160)*		
16371	15917(12047)*		
16408	16051(11532)*		
16459	16431(04849)*		
16542			
16547			
16584			
16623			
16635			
16657			
16668			
16697			
16759			
16877			
16883			
16898			
16930			
17022			
17063			
17084			
17115			
17119			
17151			
17402			
17405			
17419			
17424			
17425			
17434			
17455			
17459			
17462			

17468			
17469			
17494			
17535			
17545			
17566			
20027			
20104			
20176			
20208			
20240			
20306			
20325			
20327			
20377			
20390			
20400			
20407			
01818_M[£$]			
02154_M			
02909_M			
02977_M[$]			
03034_M			
03331_M			
03387_M			
03544_M			
03570_M			
03674_M[$]			
03687_M[£$]			
03867_M[£]			
03894_M			
04165_M			
04239_M[$]			
04374_M			
04646_M			
04681_M			
04756_M			
06437_M			
07126_M			
07684_M			
07686_M			
07996_M			
08021_M			
08041_M			

08074_M			
08090_M			
09032_M			
09085_M[$]			
09143_M			
10563_M			
10594_M			
10607_M			
10776_M			
11101_M			
11333_M			
11513_M			
11648_M			
12405_M			
12545_16364_M			
13245_M			
13435_6_M			
13975_M			
13987_M			
14020_M			
15136_M			
15714_M			
15917_M			
16018_M			
16051_M			
16111_M			
16312_M			
16431_M			
17388_M			
