# Supplementary figures and images for: The completed genome sequence of the pathogenic ascomycete fungus Fusarium graminearum
Source: BMC Genomics. 2015 Jul 22;16(1):544. doi: 10.1186/s12864-015-1756-1 (PMC4511438; doi:10.1186/s12864-015-1756-1)

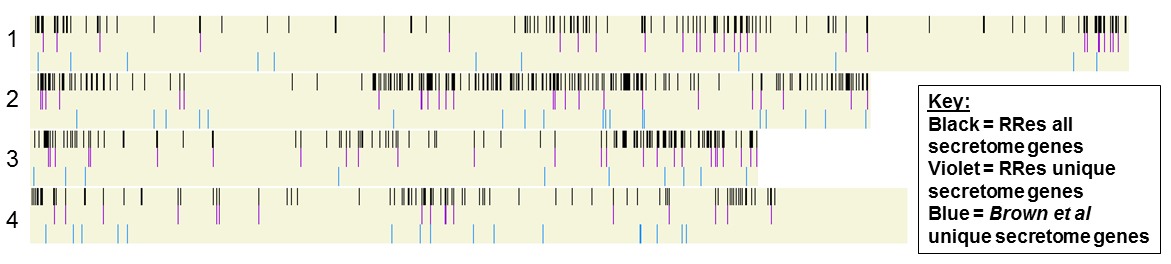

Supplement: Additional file 7: — A figure of RRes v4.0 secretome set and RRes and Brown et al. unique secretome gene prediction comparison. [file 12864_2015_1756_MOESM7_ESM.png]

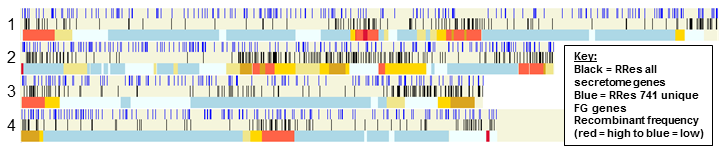

Supplement: Additional file 16: — A figure representing chromosomal locations of Fusarium graminaerum species specific gene ID’s identified in the RRes v4.0 gene annotation. [file 12864_2015_1756_MOESM16_ESM.png]

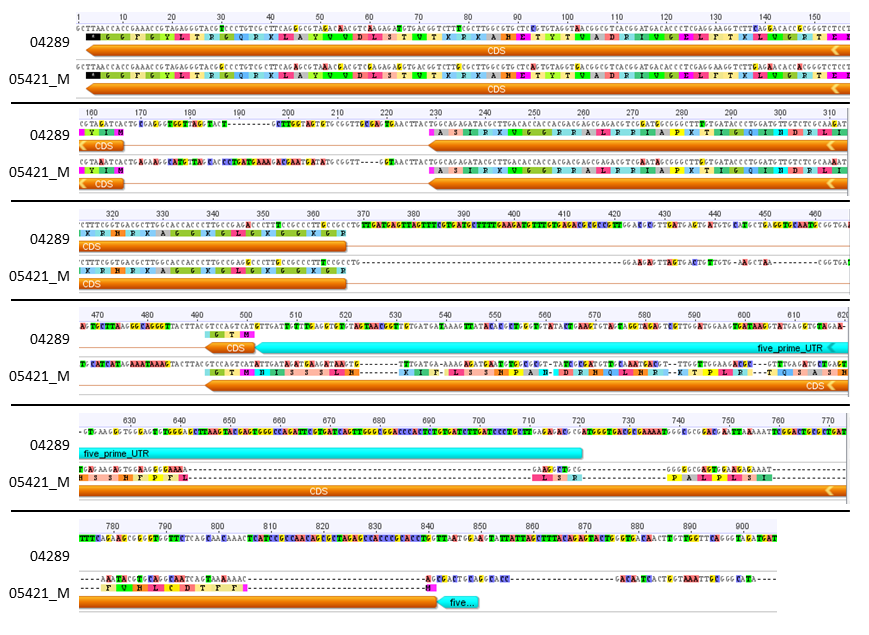

Supplement: Additional file 17: — A figure of the nucleotide alignment of genomic sequence of duplicate containing H4 protein sequences FGRRES_20411 (FGSG_04289) and FGRRES_05491_M. [file 12864_2015_1756_MOESM17_ESM.png]
