# Supplementary material for: The completed genome sequence of the pathogenic ascomycete fungus Fusarium graminearum
Source: BMC Genomics. 2015 Jul 22;16(1):544. doi: 10.1186/s12864-015-1756-1 (PMC4511438; doi:10.1186/s12864-015-1756-1)
Supplement: Additional file 8: — A table of Gene ID’s with GO term 0001071 which are identified as having nucleic acid binding transcription factor activity from either altered gene predictions or genes that were not found in the MIPS v3.2 annotation. [file 12864_2015_1756_MOESM8_ESM.pdf]

Additional file 8. Gene ID's with GO term 0001071 which are identified as having nucleic acid binding transcription factor activity from either altered gene predictions or genes that were not found in the MIPS v3.2 annotation. *Gene ID to be changed to a modified gene annotation in RRes v4.1.

Modified RRes	New RRes	New RRes blast hits
02787_M	20236 (*12631_M)	cytochrome p450
03862_M	20126	hypothetical protein FG05_30313
03873_M	20239	rta1 domain-containing protein
04170_M	20168	hypothetical protein FG05_03154
04203_M	20326	hypothetical protein FG05_35376
07575_M	20048	hypothetical protein FG05_30608
09075_M	20289	fungal transcriptional regulatory protein
09465_M	20182	hypothetical protein FG05_35086
10014_M		
10684_M		
10891_M		
10977_M		
10980_M		
11462_M		
11524_M		
12018_M		
14003_M		
15730_M		
16506_M		
16822_M		
17196_M		
17345_M		
17658_M		
12427_B_M		
