# Supplementary material for: The completed genome sequence of the pathogenic ascomycete fungus Fusarium graminearum
Source: BMC Genomics. 2015 Jul 22;16(1):544. doi: 10.1186/s12864-015-1756-1 (PMC4511438; doi:10.1186/s12864-015-1756-1)
Supplement: Additional file 9: — A table of distal centromere, possible neocentromere and telomere gene ID’s and annotations. [file 12864_2015_1756_MOESM9_ESM.pdf]

Additional file 9. Distal centromere and telomere gene ID's and annotations.

Minimum	MIPS Annotation	RRes Annotation
Centromere 1-5'		
5S_rRNA		
16043	hypothetical protein	
02778	probable histone H3 methyltransferase DIM-5	
02779	conserved hypothetical protein	
02780	probable coatomer complex beta chain	
11636	probable mago nashi protein	
11637	probable VID24 - required for vacuolar import and degradation of Fbp1p	
11632	conserved hypothetical protein	
11633	conserved hypothetical protein	
11634	related to ankyrins	
11594	related to tRNA ligase	
11595	conserved hypothetical protein	
11596	related to ribosomal protein YmL11 precursor, mitochondrial	
11597	probable 26S proteasome regulatory particle chain RPT5	
11602	probable brefeldin A resistance protein	
11603	related to KM-PA-2 protein	
16044	related to cholinesterase precursor	
centromere 1-3'		
13662	probable RPO31 - DNA-directed RNA polymerase III, 160 KD subunit	
13663	conserved hypothetical protein	
09861	conserved hypothetical protein	
09862	probable fimbrin	
09863	related to acyl-CoA cholesterol acyltransferase	
09864	related to mRNA splicing factor	
09865_M	NA	related to mitochondrial carrier protein
09866	probable ribosomal protein L18, cytosolic	
17283	related to MRM2 - Mitochondrial rRNA methyltransferase	
09868	conserved hypothetical protein	
09869	related to OST3 - oligosaccharyltransferase gamma subunit	
09870	probable CPC2 protein	
09871	conserved hypothetical protein	
09872	conserved hypothetical protein	
09873	probable glycine hydroxymethyltransferase	
17593	probable translation activator GCN1	

centromere 2 - 5'		
08944	probable glucosamine-phosphate N-acetyltransferase	
08945	conserved hypothetical protein	
08946	probable alpha-N-arabinofuranosidase / alpha-L-arabinofuranosidase	
08947	conserved hypothetical protein	
16939	related to response regulator Mcs4	
08949	conserved hypothetical protein	
08950	conserved hypothetical protein	
20155	not found	
16938_M	NA	hypothetical protein
16937	hypothetical protein	
11609_M	NA	conserved hypothetical protein
20156	not found	
11610_M	NA	conserved hypothetical protein
11590	probable TAD2 - tRNA-specific adenosine deaminase 2	
16936	hypothetical protein	
00592_M	NA	conserved hypothetical protein
13260	hypothetical protein	
centromere 2 - 3'		
16403	probable sterol glucosyltransferase	
16402	hypothetical protein	
12579	hypothetical protein	
11617	#N/A	
11618	conserved hypothetical protein	
11619	probable NADH-ubiquinone oxidoreductase 21 kDa subunit	
11622	conserved hypothetical protein	
11623	conserved hypothetical protein	
12578_M	NA	probable class E vacuolar-protein sorting and endocytosis factor
02781	probable SKI2 - antiviral protein and putative helicase	
02782	probable pyruvate dehydrogenase (lipoamide) beta chain precursor (PDB1)	
02783	probable DELTA(24)-STEROL C-METHYLTRANSFERASE (ERG6)	
012577_M	NA	related to cell cycle control protein cwf5
02784_M	NA	conserved hypothetical protein
02785	probable zuotin	
16401	probable ATP-binding cassette multidrug transport protein ATRC	
centromer		

e 3 - 5'		
16651_M	related to CECR1 protein	
06423	conserved hypothetical protein	
06424	related to SRP40 - suppressor of mutant AC40 of RNA polymerase I and III	
06425	conserved hypothetical protein	
12916_M	NA	related to probable gtp-binding protein engb
16652	hypothetical protein	
16653	related to ubiquitin-protein ligase	
06427	related to YBR267w	
06428	conserved hypothetical protein	
centromere 3 - 3'		
13936	hypothetical protein	
11598	related to L-fucose permease	
17530	related to neurofibromin	
10716	related to dna exoribonuclease Dhp1p	
10717	related to transport protein USO1	
10718	related to protein arginine N-methyltransferase 3	
17529	related to permeases - unknown function	
13934	related to RAV1 - similarity to Drosophila DmX gene	
17739	conserved hypothetical protein	
10721	conserved hypothetical protein	
10722	probable isoleucine--tRNA ligase	
10723	probable hymA gene	
10724	related to SWI/SNF complex 60 kDa subunit	
centromere 4- 5'		
07954	related to mouse proteinase activated receptor 2	
07955	related to GPI-anchor transamidase GAA1	
07956	related to 26S proteasome regulatory particle chain RPN9	
13257	probable NADH2 dehydrogenase (ubiquinone) 40K chain	
13258	probable NADH2 dehydrogenase (ubiquinone) 40K chain	
07958	related to ubiquitin-specific protease 22	
07959	conserved hypothetical protein	
07960	related to YTP1	
07961	conserved hypothetical protein	
07962	probable Rad54 homolog MUS-25	
16933	hypothetical protein	
07963	related to ornithine transporter 2	
07964	related to member of RSC complex	

16934	hypothetical protein	
07966	probable transmembrane protein UsgS	
07967	conserved hypothetical protein	
16935	hypothetical protein	
13259	hypothetical protein	
07969	conserved hypothetical protein	
07970_M	NA	NONE
centromere 4- 3'		
11628	conserved hypothetical protein	
11627	probable HTA2 - histone H2A.2	
11626	probable HTB1 - histone H2B	
13661	conserved hypothetical protein	
17282_M	NA	related to ATG4 - essential for autophagocytosis
09857	related to dnaJ-like proteins	
09856	conserved hypothetical protein	
09855	related to O-succinylhomoserine (thiol)-lyase	
17281	hypothetical protein	
09853	conserved hypothetical protein	
09852	conserved hypothetical protein	
09851	probable SAP1 - member of the AAA-protein family	
09850	related to COG6 - conserved oligomeric golgi complex	
13660	probable APG9 - integral membrane protein required for Cvt and autophagy transport	
13659	hypothetical protein	

Telomeres -first 2 genes			
	ID		
chr 1 - 5'	16072_M	hypothetical protein	homology in chr2 ?
	11606	related to hexose transporter protein	
chr 1 - 3'	17444	related to flavin-containing monooxygenase	
	13822_M	related to flavin-containing monooxygenase	
chr 2 - 5'	14031_M	conserved hypothetical protein	no homology in other chr
	15663	hypothetical protein	

chr 2 - 3'	11586	conserved hypothetical protein	
	11587_M	probable sterol glucosyltransferase	no homology in other chr
chr 3 - 5'	20223	NA	
	12581_2_M	hypothetical protein	NA but MIPS given;
chr 3 - 3'			
	15657_M	hypothetical protein	
	20327	NA	
	20328	NA	Has homology to chromosome 2 -5' end !
chr 4 - 5'	14027_16070_11614_M	Ankyrin repeat domain protein; amily: Ank_2 (PF12796)	
	11613_M	conserved hypothetical protein	
	16071	hypothetical protein	
chr 4 - 3'	tRNA		
(defense response related)	08956_M	NB-ARC doamin signalling motif; related to kinesin light chain	
	13478	conserved hypothetical protein	
	08955_M	related to WD40-repeat protein (notchless protein)	
	8954	conserved hypothetical protein	
	13477	conserved hypothetical protein	
	13476	conserved hypothetical protein	
	17133	related to RSA4 - WD-repeat protein required for maturation and efficient intra-nuclear transport or pre-60S ribosomal subunits	NA but MIPS given
(defense response related)	17709_M	NB-ARC domain signalling motif; related to calcium-independent phospholipase A2	
	20409	NA	
	20412	NA	
	rRNA cluster	28S(?)rRNA	

neocentromere			
	ID		
chr 3 - 5’			
	17445	Aconitase/3-isopropylmalate dehydratase	iron
	13824	P-loop containing nucleoside triphosphate hydrolase, NACHT	
	17446	Multi-copper oxidase	copper
	11236	phosphoesterase	
	17447	NACHT domain	
	11234_M	TRANSCRIPTION TERMINATION FACTOR 2	
	11232	Amine oxidase	
	11231	MOLYBDOPTERIN COFACTOR SULFURASE	
chr 3 - 3’			
	11620	ARRESTIN-RELATED TRAFFICKING ADAPTER 3-RELATED	
	17589	Marvel domain, MAL and related proteins for vesicle trafficking and membrane link	
	17597_11239_M	Potassium channel	
	11240	ATP-BINDING CASSETTE TRANSPORTER	
	11241	EXOPOLYPHOSPHATASE	
	17587	Aerobactin siderophore biosynthesis	iron
