# Supplementary material for: The completed genome sequence of the pathogenic ascomycete fungus Fusarium graminearum
Source: BMC Genomics. 2015 Jul 22;16(1):544. doi: 10.1186/s12864-015-1756-1 (PMC4511438; doi:10.1186/s12864-015-1756-1)
Supplement: Additional file 10: — A table of the alignment positions of flanking sequences of AT rich regions from the F. verticillioides chromosomes to the sequences flanking the centromeres in RRes v4.0 F. graminearum . [file 12864_2015_1756_MOESM10_ESM.pdf]

Additional file 10. The alignment positions of the AT rich regions from the *F. verticillioides* chromosomes to the sequences immediately flanking the centromeres in RRes v4.0 *F. graminearum*.

FG Chromosome centromere	Amino centromere flank			Carboxyl centromere flank		
	Chromosome	Position (Mbp)	Orientation (R/F)*	Chromosome	Position (Mbp)	Orientation (R/F)*
1	5	2.65	F	5	0.00	F
2	6	3.90	F	N/A	N/A	N/A
3	2	2.06	F	DS486012 (supercontig)	0.00	R
4	4	3.30	F	4	2.30	R
4 (neocentromere)	N/A	N/A	N/A	N/A	N/A	N/A

*R/F = reverse complement or forward orientation of reference sequence
**N/A = no homology
