# Supplementary material for: The completed genome sequence of the pathogenic ascomycete fungus Fusarium graminearum
Source: BMC Genomics. 2015 Jul 22;16(1):544. doi: 10.1186/s12864-015-1756-1 (PMC4511438; doi:10.1186/s12864-015-1756-1)
Supplement: Additional file 11: — A table of RRes v4.0 gene models that have changed since MIPS v3.2 that are present in PHI-base. [file 12864_2015_1756_MOESM11_ESM.pdf]

Additional file 11. RRes v4.0 gene models that have changed since MIPS v3.2 that are present in PHI-base. [A] MIPS gene ID version 30 unless [B] MIPS gene call version 3.2; [C] gene homolog name provided in brackets.

PHI-base ID	MIPS gene ID [A]	Mutant phenotype in planta	Gene name or homolog[C]
PHI:1182	FGSG_13318	Reduced virulence	Sc Mec1
PHI:1206	FGSG_04053	Reduced virulence	Sp Prp4
PHI:1251	FGSG_01506 [B]	Unaffected pathogenicity	
PHI:1270	FGSG_03146 [B]	Unaffected pathogenicity	
PHI:1276	FGSG_09150 [B]	Unaffected pathogenicity	
PHI:1278	FGSG_11614 [B]	Unaffected pathogenicity	
PHI:1281	FGSG_02153 [B]	Unaffected pathogenicity	
PHI:1286	FGSG_07812 [B]	Unaffected pathogenicity	
PHI:1338	FGSG_13313	Unaffected pathogenicity	GzbZIP020
PHI:1339	FGSG_13759	Unaffected pathogenicity	GzbZIP021
PHI:1359	FGSG_02788	Unaffected pathogenicity	GzC2H019
PHI:1377	FGSG_06168	Unaffected pathogenicity	GzC2H040
PHI:1385	FGSG_07075	Unaffected pathogenicity	GzC2H048
PHI:1386	FGSG_07187	Unaffected pathogenicity	GzC2H049
PHI:1392	FGSG_07751	Unaffected pathogenicity	GzC2H055
PHI:1397	FGSG_07952	Unaffected pathogenicity	GzC2H060
PHI:1400	FGSG_08246	Unaffected pathogenicity	GzC2H063
PHI:1431	FGSG_12970	Unaffected pathogenicity	Pac1
PHI:1432	FGSG_12973	Unaffected pathogenicity	GzC2H100
PHI:1435	FGSG_13492	Unaffected pathogenicity	GzC2H104
PHI:1437	FGSG_13896	Unaffected pathogenicity	GzC2H106
PHI:1466	FGSG_07087	Unaffected pathogenicity	GzCCAAT006
PHI:1483	FGSG_04557	Unaffected pathogenicity	GzHMG015
PHI:1486	FGSG_05604	Unaffected pathogenicity	GzHMG018
PHI:1498	FGSG_10762	Lethal	GzHMG030

PHI:1502	FGSG_12323	Unaffected pathogenicity	GzHMG034
PHI:1527	FGSG_13431	Lethal	GzHOMEL040
PHI:1627	FGSG_08551	Unaffected pathogenicity	GzTF2S002
PHI:1645	FGSG_07433	Unaffected pathogenicity	GzWing017
PHI:1660	FGSG_11696	Unaffected pathogenicity	GzCCCH004
PHI:1669	FGSG_10277	Unaffected pathogenicity	GzCCHC009
PHI:1675	FGSG_06542	Unaffected pathogenicity	GzDHHC003
PHI:1684	FGSG_04258	Unaffected pathogenicity	GzRad002
PHI:1695	FGSG_10977	Unaffected pathogenicity	GzZC010
PHI:1696	FGSG_11051	Unaffected pathogenicity	GzZC011
PHI:1697	FGSG_11462	Unaffected pathogenicity	GzZC012
PHI:1698	FGSG_10891	Unaffected pathogenicity	GzZC013
PHI:1701	FGSG_04496	Unaffected pathogenicity	GzZC016
PHI:1702	FGSG_07575	Unaffected pathogenicity	GzZC017
PHI:1707	FGSG_04170	Unaffected pathogenicity	GzZC022
PHI:1713	FGSG_02825	Unaffected pathogenicity	GzZC028
PHI:1714	FGSG_06448	Lethal	GzZC029
PHI:1715	FGSG_06380	Unaffected pathogenicity	GzZC030
PHI:1718	FGSG_13652	Unaffected pathogenicity	GzZC033
PHI:1721	FGSG_13344	Unaffected pathogenicity	GzZC036
PHI:1748	FGSG_12528	Unaffected pathogenicity	GzZC063
PHI:1751	FGSG_09726	Unaffected pathogenicity	GzZC066
PHI:1805	FGSG_08028	Reduced virulence	GzZC120
PHI:1823	FGSG_09075	Unaffected pathogenicity	GzZC138
PHI:1826	FGSG_10980	Unaffected pathogenicity	GzZC141
PHI:1833	FGSG_04203	Unaffected pathogenicity	GzZC148
PHI:1838	FGSG_02787	Unaffected pathogenicity	GzZC153
PHI:1840	FGSG_06460	Unaffected pathogenicity	GzZC155
PHI:1846	FGSG_12729	Unaffected pathogenicity	GzZC161
PHI:1851	FGSG_09064	Lethal	GzZC166
PHI:1863	FGSG_04671	Unaffected pathogenicity	GzZC178

PHI:1864	FGSG_14003	Unaffected pathogenicity	GzZC179
PHI:1865	FGSG_13455	Unaffected pathogenicity	GzZC180
PHI:1886	FGSG_07693	Unaffected pathogenicity	GzZC201
PHI:1894	FGSG_05716	Unaffected pathogenicity	GzZC209
PHI:1905	FGSG_03294	Unaffected pathogenicity	GzZC220
PHI:1908	FGSG_03226	Unaffected pathogenicity	GzZC223
PHI:1911	FGSG_03207	Unaffected pathogenicity	GzZC226
PHI:1949	FGSG_03485	Lethal	GzZC264
PHI:1957	FGSG_04888	Unaffected pathogenicity	GzZC272
PHI:1963	FGSG_06503	Unaffected pathogenicity	GzZC278
PHI:1995	FGSG_00196	Unaffected pathogenicity	GzZC310
PHI:2386	FGSG_12857	Loss of pathogenicity	ACL1
PHI:2395	FGSG_00950	Reduced virulence	GzSYN1
