# Supplementary material for: The completed genome sequence of the pathogenic ascomycete fungus Fusarium graminearum
Source: BMC Genomics. 2015 Jul 22;16(1):544. doi: 10.1186/s12864-015-1756-1 (PMC4511438; doi:10.1186/s12864-015-1756-1)
Supplement: Additional file 12: — A table of RRes v4.0 F. graminearum recombinant and non-recombinant region comparison. [file 12864_2015_1756_MOESM12_ESM.pdf]

Additional file 12. RRes *F. graminearum* recombinant and non-recombinant region comparison. No observable pattern was identified between the high recombinant and non-highly recombinant regions when chromosomal positions were also taken into account.

Region	Chrom	Position	length	AT (%)	Gene no.	Gene no. per 100 kb	Secretome no.	Modified Gene no.	New gene no.
Recombinant region 1	1 (5' long arm)	32,785 575,729	542,944	53.4	233	42.91	20	26	7
Recombinant region 2	3 (5' long arm)	12,014 401,069	389,055	53.6	169	43.44	18	22	8
Recombinant region 3	1 (central long arm)	5,639,082 6,032,689	393,607	52.4	161	40.90	13	20	2
Recombinant region 4	2 (3'end long arm)	8,387,552 8,806,410	418,858	53.7	171	40.83	18	33	3
Recombinant region 5	4 (central long arm)	4,064,366 4,608,192	543,826	53.2	249	45.79	19	37	8
Non-recombinant region 1	1 (adjacent to 5' end long arm)	866,719 1,471,900	605,181	50.9	221	36.52	2	13	5
Non-recombinant region 2	3 (adjacent to 5' end long arm)	651,956 1,687,347	1,035,391	51.4	419	40.47	7	28	13
Non-recombinant region 3	4 (3' end short arm)	7,219,363 7,842,145	622,782	52.4	263	42.23	18	46	9
Non-recombinant region 4	2 (central short arm)	1,757,141 2,235,287	478,146	51.2	170	35.55	0	29	4
