# Supplementary material for: The completed genome sequence of the pathogenic ascomycete fungus Fusarium graminearum
Source: BMC Genomics. 2015 Jul 22;16(1):544. doi: 10.1186/s12864-015-1756-1 (PMC4511438; doi:10.1186/s12864-015-1756-1)
Supplement: Additional file 13: — A description of RRes repeat masking details and a table of gene ID’s identified with transposon domains. [file 12864_2015_1756_MOESM13_ESM.pdf]

Additional file 13. RRes Repeat masking details and gene ID's identified with transposon domains.

In total using RepeatModeller, 12 repeat motif containing sequences were identified including a long terminal direct repeat (LTR) of the gypsy group, TcMar-Ant1, TcMar-Pogo, TcMar-Marin and TcMar-Fot1 transposon identified as two large repeat sequences, 15 other unknown repeat sequences, one SINE and one rRNA located in the chromosomal 4 repetitive region (previously discussed).

Transposon Gene ID (FGRRES_)	Transposon class
08954	TcMar-Ant1
06490	TcMar-Ant1, TcMar-ISRm1
13478	TcMar-Ant1, LTR-Gypsy
05781	TcMar-Tc1
15950	TcMar-Fot1
17263	TcMar-Fot1
16975	TcMar-Fot1
13431_M	TcMar-Fot1
07243	TcMar-Pogo
05264	TcMar-Pogo
08846	TcMar-Marin
20409	LTR/Gypsy
