# Supplementary material for: The completed genome sequence of the pathogenic ascomycete fungus Fusarium graminearum
Source: BMC Genomics. 2015 Jul 22;16(1):544. doi: 10.1186/s12864-015-1756-1 (PMC4511438; doi:10.1186/s12864-015-1756-1)
Supplement: Additional file 14: — A table of transposon and repeat content of centromeres and subtelomeric AT rich regions. [file 12864_2015_1756_MOESM14_ESM.pdf]

Additional file 14. Transposon and repeat content of centromeres and subtelomeric AT rich regions.

Chromosome/region	Simple repeat (total length bp) no.	Low complexity repeat (total length bp) no.	Transposon (total length bp) no.	Length of region (bp)	Transposon classes
1 Amino subtelomere	(69) 1	0	(1,725) 1	6,000	MarCry-1_FO
1 Centromere	(854) 17	(586) 9	(31,828) 34	56,581	MarCry-1_FO, LTR-Gypsy
1 Carboxyl subtelomere	(1,744) 38	(630) 8	(141)	15,700	MarCry-1_FO
2 Amino subtelomere	(60) 1	0	(3,738) 2	6,000	MarCry-1_FO
2 Centromere	(1,848) 33	(442) 8	(35,049) 43	65,181	MarCry-1_FO, LTR-Gypsy
2 Carboxyl subtelomere	(123) 2	0	(1,159) 3	3,600	MarCry-1_FO, LTR-Gypsy
3 Amino subtelomere	(384) 7	(42) 1	(746) 2	10,800	MarCry-1_FO, LTR-Gypsy
3 Centromere	(278) 7	0	(35,251) 25	56,387	MarCry-1_FO, LTR-Gypsy
3 Carboxyl subtelomere	(1,003) 18	(250) 5	(6,080) 12	21,300	MarCry-1_FO, LTR-Gypsy
3 neocentromere	(513) 10	(679) 2	(7,901) 10	14,951	MarCry-1_FO, LTR-Gypsy
4 Amino subtelomere	0	0	(372) 1	661	LTR-Gypsy
4 centromere	(746) 16	(228) 5	(33,842) (31)	60,933	MarCry-1_FO, LTR-Gypsy
