# Supplementary material for: The completed genome sequence of the pathogenic ascomycete fungus Fusarium graminearum
Source: BMC Genomics. 2015 Jul 22;16(1):544. doi: 10.1186/s12864-015-1756-1 (PMC4511438; doi:10.1186/s12864-015-1756-1)
Supplement: Additional file 18: — A table of RRes v4.0 all genes, secretome subset genes, and unique gene sets per chromosome arm. [file 12864_2015_1756_MOESM18_ESM.pdf]

Additional file 18. RRes *F. graminearum* detailed statistics of chromosome arms.

Chromosome	Genes	FG unique	Secretome	GC (%)	CENTROMERE	Genes	FG unique	Secretome	GC (%)	Total GC content (%)*
1	3,354	143	111	48.5		1,039	66	34	48.2	48.3
2	1,250	99	42	48.1		2,395	130	192	47.8	47.6
3	2,104	102	61	48.6		986	58	81	47.6	48.0
4	1,937	119	61	48.4		1,095	48	34	49.7<br>48.6**	48.8
Total	8,645	463	275	48.4		5,515	302	341	48.3<br>48.1**	48.2 (Avg.)

*Excluding N bases

** Excluding large repetitive sequence at the carboxyl end of chromosome 4

This table excludes the 4 unique genes found on supercontig 3.12
