# Supplementary material for: The completed genome sequence of the pathogenic ascomycete fungus Fusarium graminearum
Source: BMC Genomics. 2015 Jul 22;16(1):544. doi: 10.1186/s12864-015-1756-1 (PMC4511438; doi:10.1186/s12864-015-1756-1)

Additional file 19. The composition of the centromere sequences in chromosomes 1-4 and read coverage.

Summary frequency of bases, percentage composition and percentage GC content for each of the centromeres of chromosomes 1-4.

	1		2		3		4	
Composition	Freq	%	Freq	%	Freq	%	Freq	%
A:	24,365	43.10%	29,288	44.90%	23,774	42.20%	27,334	44.90%
C:	3,661	6.50%	4,594	7.00%	3,523	6.20%	3,643	6.00%
G:	3,588	6.30%	4,277	6.60%	3,722	6.60%	3,754	6.20%
T:	24,955	44.10%	27,022	41.50%	25,368	45.00%	26,202	43.00%
N:	12	0.00%	0	0.00%	0	0.00%	0	0.00%
GC:	7,249	12.80%	8,871	13.60%	7,245	12.80%	7,397	12.10%

Image of mapped reads to centromere sequences (75 kbp window). Green = A, Blue = T, Yellow = C, Red = G.

Centromere1

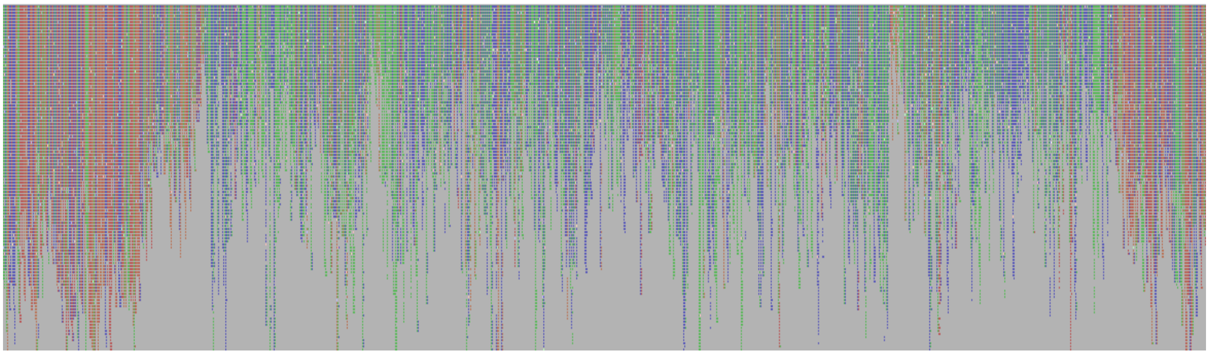

Centromere2

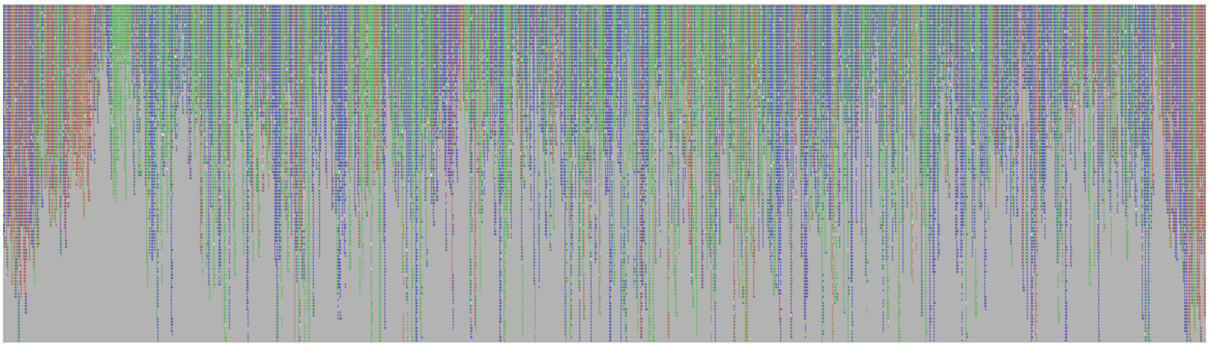

Centromere3

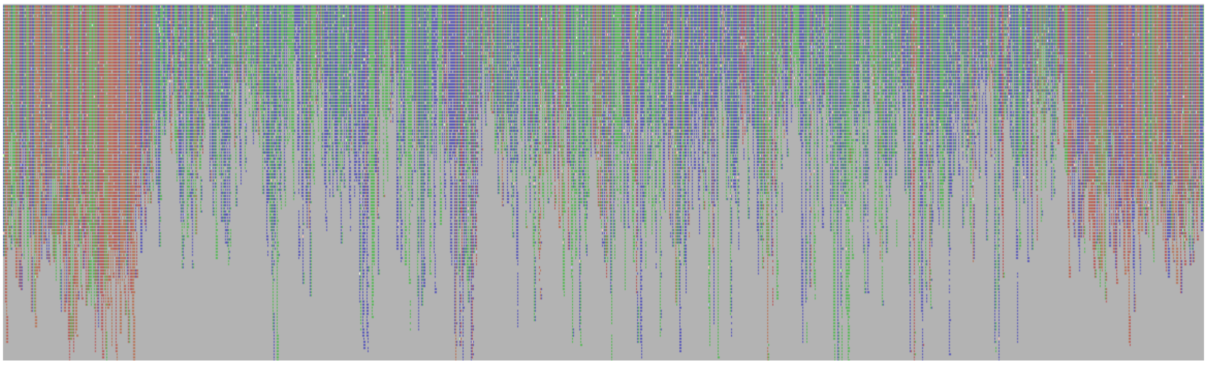

Centromere4

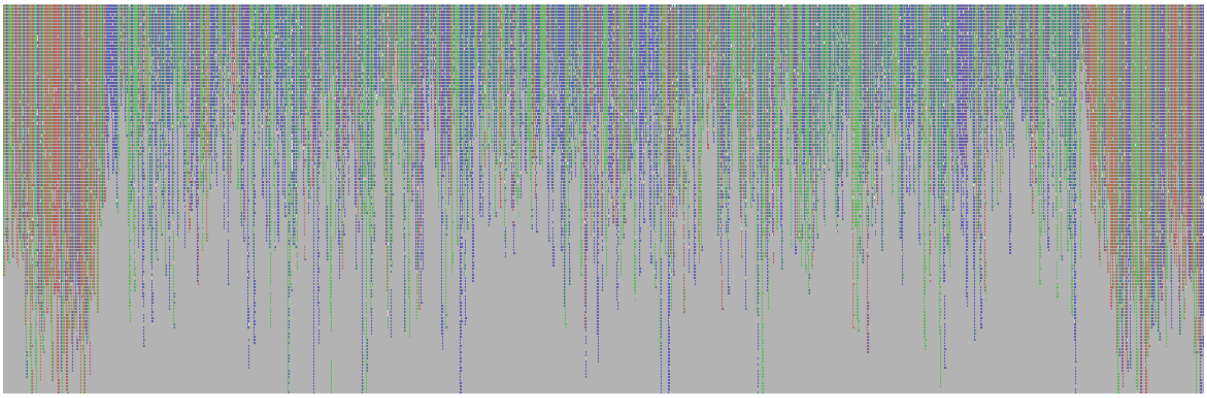

Image of Chromosome regions GC/AT graphs. Yellow represents the proposed centromere sequences and the region annotated with a red arrow represent regions of lower AT content and higher GC. Region 1 of chromosome 2 and region 3 of chromosome 3 represents a region of respectively approximately 7,194 bp and 450 bp and 22.8% and 53.8% GC content.

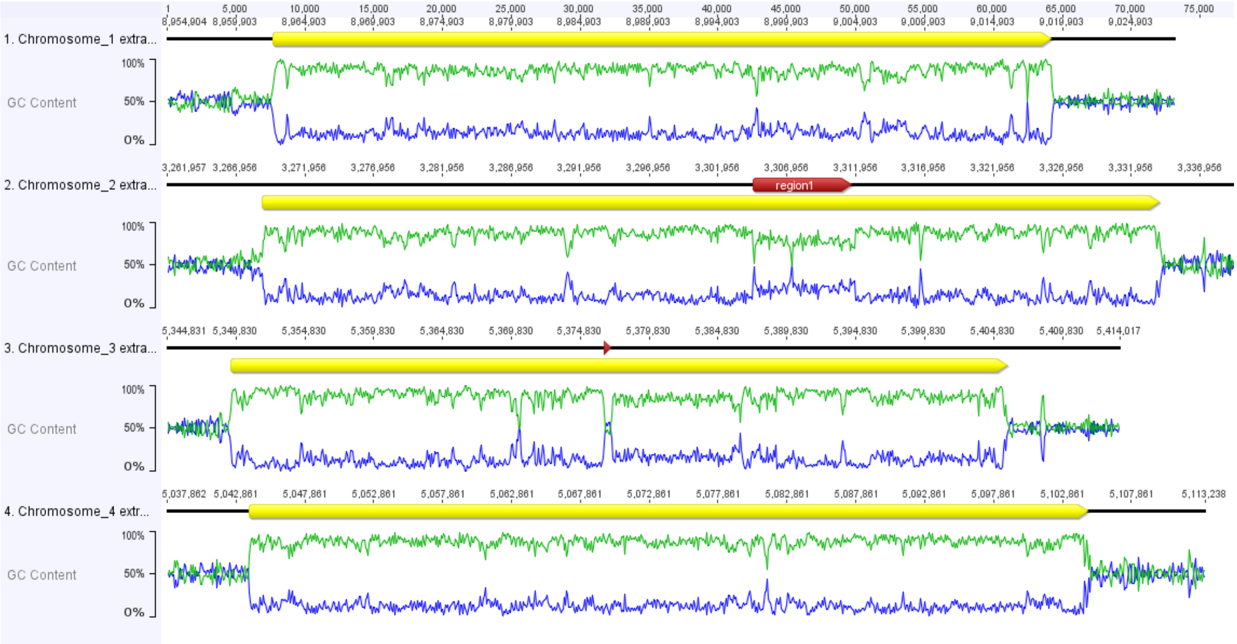

Supplement: Additional file 19: — Shows the mapped read coverage across the centromeres and the corresponding GC content. [file 12864_2015_1756_MOESM19_ESM.pdf]
