# Supplementary material for: The completed genome sequence of the pathogenic ascomycete fungus Fusarium graminearum
Source: BMC Genomics. 2015 Jul 22;16(1):544. doi: 10.1186/s12864-015-1756-1 (PMC4511438; doi:10.1186/s12864-015-1756-1)
Supplement: Additional file 20: — A set of instructions for how to stay updated and contribute to F. graminearum PH-1 gene models changes prior to future Ensembl Fungi version releases. [file 12864_2015_1756_MOESM20_ESM.pdf]

Additional file 20. Instructions to join the mailing list for the *F. graminearum* RRes v4.1 annotation. Subscribers can submit new or corrected gene models and receive updated annotation versions via custom tracks in Ensembl Fungi.

<u>Joining the mailing list:</u>

Step 1: Copy the text into your browser "https://www.lists.rothamsted.ac.uk/mailman/listinfo/fgrresv41"

Step2: In the "Subscribing to Fgrresv41" section fill in your name, email address and password.

Step3: A confirmation email will be sent to you. Click the confirmation link. Please check spam folder and promotional folder if using gmail.

Step4: To post a message use this email address "fgrresv41@lists.rothamsted.ac.uk". Postings may appear in the forum tab of a gmail account.

<u>Submission of gene models new or curated:</u>

Step 1: send a gff3 file to the mailing group or within the email (if only 1-10 gene models) of your gene models to be curated by the admin. Include in your message any supporting evidence to justify the change. Please see example gff3 below which shows two exons of the gene 11979 on chromosome 1 which is the strand, and the locus_tag which is the name of the gene.

1	.	CDS	5340879	5342780	.	-	.	locus_tag=11979_M;ID=RK1
1	.	CDS	5339808	5340830	.	-	.	locus_tag=11979_M;ID=RK1

The administrator will then transfer the gene model or curate an existing entry to the mailing site set and include in an updated custom track URL for Ensembl Fungi for all users to see. After approx. 6 months periodically an update will be submitted to ENA and subsequently Ensembl Fungi.
